# Down-Top Strategy Engineered Large-Scale Fluorographene/PBO Nanofibers Composite Papers with Excellent Wave-Transparent Performance and Thermal Conductivity

**DOI:** 10.1007/s40820-025-01878-y

**Published:** 2025-08-20

**Authors:** Yuhan Lin, Lin Tang, Mingshun Jia, Mukun He, Junliang Zhang, Yusheng Tang, Junwei Gu

**Affiliations:** 1 Shaanxi Key Laboratory of Macromolecular Science and Technology, School of Chemistry and Chemical Engineering, Northwestern Polytechnical University, Xi’an, 710072 People’s Republic of China; 2 Chongqing Key Laboratory of Green Catalysis Materials and Technology, College of Chemistry, Chongqing Normal University, Chongqing, 401331 People’s Republic of China

**Keywords:** PBO nanofibers, Fluorinated graphene, Wave-transparency, Thermal conductivity

## Abstract

With the miniaturization and high-frequency evolution of antennas in 5G/6G communications, aerospace, and transportation, polymer composite papers integrating superior wave-transparent performance and thermal conductivity for radar antenna systems are urgently needed. Herein, a down-top strategy was employed to synthesize poly(p-phenylene benzobisoxazole) precursor nanofibers (*pre*PNF). The *pre*PNF was then uniformly mixed with fluorinated graphene (FG) to fabricate FG/PNF composite papers through consecutively suction filtration, hot-pressing, and thermal annealing. The hydroxyl and amino groups in *pre*PNF enhanced the stability of FG/*pre*PNF dispersion, while the increased π-π interactions between PNF and FG after annealing improved their compatibility. The preparation time and cost of PNF paper was significantly reduced when applying this strategy, which enabled its large-scale production. Furthermore, the prepared FG/PNF composite papers exhibited excellent wave-transparent performance and thermal conductivity. When the mass fraction of FG was 40 wt%, the FG/PNF composite paper prepared via the down-top strategy achieved the wave-transparent coefficient (|*T*|^2^) of 96.3% under 10 GHz, in-plane thermal conductivity (*λ*_*∥*_) of 7.13 W m^−1^ K^−1^, and through-plane thermal conductivity (*λ*_⊥_) of 0.67 W m^−1^ K^−1^, outperforming FG/PNF composite paper prepared by the top-down strategy (|*T*|^2^ = 95.9%, *λ*_*∥*_ = 5.52 W m^−1^ K^−1^, *λ*_⊥_ = 0.52 W m^−1^ K^−1^) and pure PNF paper (|*T*|^2^ = 94.7%, *λ*_*∥*_ = 3.04 W m^−1^ K^−1^, *λ*_⊥_ = 0.24 W m^−1^ K^−1^). Meanwhile, FG/PNF composite paper (with 40 wt% FG) through the down-top strategy also demonstrated outstanding mechanical properties with tensile strength and toughness reaching 197.4 MPa and 11.6 MJ m^−3^, respectively.

**Supplementary Information:**

The online version contains supplementary material available at 10.1007/s40820-025-01878-y.

## Highlights

- The down-top strategy enables large-scale production of poly(p-phenylene benzobisoxazole) nanofiber (PNF) paper with excellent intrinsic wave-transparent performance, thermal conductivity, and mechanical strength while significantly reduces the preparation time and cost.
- Fluorinated graphene (FG)/PNF composite papers exhibit superior wave-transparent performance and thermal conductivity. When the mass fraction of FG is 40 wt%, its |*T*|² reaches 96.3% under 10 GHz while* λ*_∥_ and *λ*_⊥_ increase to 7.13 and 0.67 W m^-1^ K^-1^, respectively.
- FG/PNF composite paper with 40 wt% of FG also displays excellent mechanical properties, with the tensile strength and toughness reaching 197.4 MPa and 11.6 MJ m^-3^, respectively.

**Supplementary Information:**

The online version contains supplementary material available at 10.1007/s40820-025-01878-y.

## Graphical abstract

**Figure:**
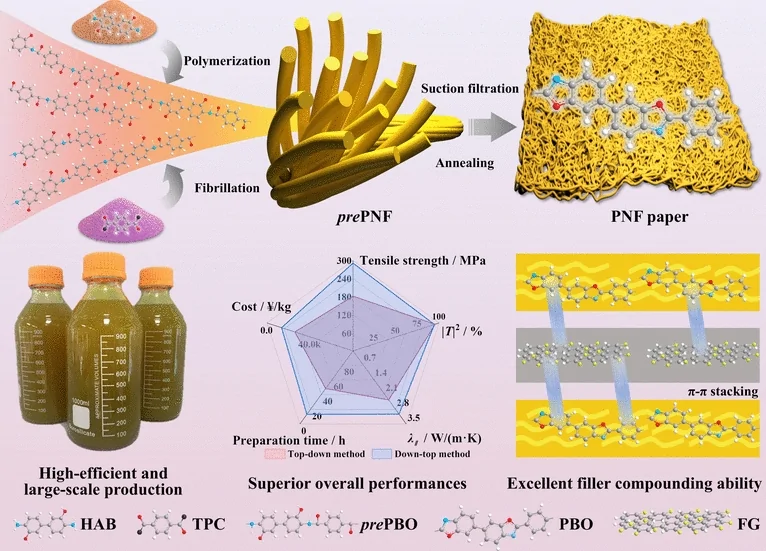

## Introduction

The miniaturization, integration, and high-frequency evolution of electronic components in 5G/6G communications, aerospace, and transportation has resulted in complex electromagnetic environments and heat accumulation challenges [1–3]. Under high power density, heat accumulation will reduce the wave-transparent performance of devices, shorten service life, and even cause safety hazards [4]. Therefore, materials combining superior wave-transparency and thermal conductivity are increasingly demanded in fields of mobile terminal antennas, airborne electromagnetic countermeasure equipment, and vehicular radar [5, 6]. Poly(p-phenylene benzobisoxazole) nanofiber (PNF) paper exhibits low dielectric constant (*ε*) and dielectric loss tangent (tan*δ*), which shows high wave-transparent performance [7, 8]. Owing to the highly oriented molecular chains and high crystallinity, PNF paper demonstrates significantly enhanced thermal conductivity compared to conventional wave-transparent polymer matrices (e.g., epoxy resin, cyanate ester, aramid paper), making it a promising candidate for high-performance materials combining with superior wave transparency and thermal conductivity [9–11].

Currently, PNF paper is primarily fabricated via two approaches: spinning strategy and dissolution of poly(p-phenylene benzobisoxazole) (PBO) fibers [12]. Spinning strategy primarily involves obtaining PNF through electrospinning or solution spinning in a down-top (or bottom-up) strategy, which was then fabricated into PNF paper [13, 14]. For instance, Liu et al. [15] prepared PNF paper via “electrospinning-heat treatment” using *p*-nitrobenzoyl chloride (*p*NBC), 2,2-bis(3-amino-4-hydroxyphenyl)hexafluoropropane (6FAP), and hexafluorodianhydride (6FDA). The thermal decomposition temperature of obtained PNF paper was reduced to 509 °C although it exhibited a low dielectric constant (*ε*) of 1.64 at 1 MHz. Li et al. [16] prepared polyamide benzoxazole (PABO) from 2-(3-aminophenyl)-benzoxazol-5-amine (APBOA) and terephthaloyl chloride (TPC), which was then sheared into deionized water to form PNF. The layered PNF paper fabricated by vacuum-assisted filtration and hot-pressing achieved a tensile strength of 199.3 MPa and thermal decomposition temperature of 550 °C. Nevertheless, the introduction of heteroatoms and amide bonds in PNF paper degrades thermal stability and mechanical properties compared to pristine PBO fibers. Furthermore, the complexity of electrospinning also restricts large-scale production [12, 17]. Alternatively, the top-down strategy is by dissolving PBO fibers under strong acidic/alkaline conditions to yield PNF [18, 19], which is subsequently processed into PNF paper via vacuum filtration, blade coating, or sol–gel-film conversion [20]. This approach achieves simultaneous low *ε*, tan*δ*, and high thermal conductivity [21, 22]. For instance, Yang et al. [23] dissolved PBO fibers in a mixed acid of trifluoroacetic acid (TFA) and methanesulfonic acid (MSA), and then fabricated PNF paper through deprotonation and vacuum filtration. Although low *ε* (< 3.0) and tan*δ* (< 0.5) were achieved, the through-plane thermal conductivity (*λ*_⊥_) and tensile strength was only 0.05 W m^−1^ K^−1^ and 65.4 MPa, respectively. In our previous work [7], protonic acid was utilized to dissolve PBO fibers and the inter-PNF interactions were enhanced via sulfate coordination. The obtained PNF paper demonstrated superior wave-transparent performance (|*T*|^2^ = 95.9% at 1 MHz), thermal conductivity (*λ*_*∥*_ = 2.46 W m^−1^ K^−1^, *λ*_⊥_ = 0.17 W m^−1^ K^−1^), thermal stability (decomposition temperature of 640 °C), and tensile strength (254.6 MPa). These results confirm that the top-down strategy can produce wave-transparent and thermally conductive PNF paper with well-balanced properties. However, strong protonic acids (e.g., MSA and TFA) have to be applied for dissolution due to the chemical inertness of PBO molecules, causing severe equipment corrosion. Furthermore, high costs and low efficiency further impede the industrial-scale applications of this strategy [24].

To further enhance the wave-transparent and thermal conduction performances of PNF paper, researchers commonly incorporate fillers with low *ε* and high thermal conductivity, such as boron nitride nanosheets (BNNS) [25, 26] and fluorinated graphene (FG) [27, 28]. However, the chemical inertness of PBO molecules leads to weak compatibility and interactions between PNFs or fillers and PNFs. This weak interfacial bonding enhances the polarization of various molecules and crystal structures within the composite paper through orientation-disorientation processes caused by the external electromagnetic field. Additionally, interfacial thermal resistance causes phonon scattering. Thereby, wave-transparent and thermal conduction performances would be degraded [29, 30]. Currently, the interaction and compatibility between fillers and PNFs were commonly enhanced through surface functionalization of fillers [10, 31]. Huang et al. [32] modified BNNS using oxalyl chloride to generate negatively charged BNNS (M-BNNS). The dispersion of M-BNNS in PNF acid solution was enhanced, and its interaction with PNF was strengthened via electrostatic forces. When the mass fraction of M-BNNS was 10 wt%, the M-BNNS/PNF composite paper exhibited the lowest *ε* (2.9) and tan*δ* (0.01), along with superior thermal conductivity (*λ*_*∥*_ = 21.34 W m^−1^ K^−1^), outperforming the composite paper containing unmodified BNNS. In our previous work, Gu et al. [7] functionalized boron nitride with benzidine (*m*-BN) to establish hydrogen bonding and π-π interactions with PNF. The *m*-BN/PNF paper exhibited enhanced *λ*_*∥*_ (9.68 W m^−1^ K^−1^) and *λ*_⊥_ (0.84 W m^−1^ K^−1^), exceeding unmodified BN/PNF paper (*λ*_*∥*_ = 7.72 W m^−1^ K^−1^, *λ*_⊥_ = 0.71 W m^−1^ K^−1^). Concurrently, it exhibited a lower *ε* (3.55) and tan*δ* (0.033) compared to the unmodified BN/PNF composite paper (*ε* = 3.76, tan*δ* = 0.042). Evidently, enhancing the electrostatic and π-π interactions between fillers and PNFs through surface modification can effectively reduce polarization phenomena and phonon scattering, thereby improving the wave-transparent and thermal conduction capabilities of the composite paper. However, the process of filler surface functionalization is relatively complex and challenging for large-scale production [24].

**Fig. 1:**
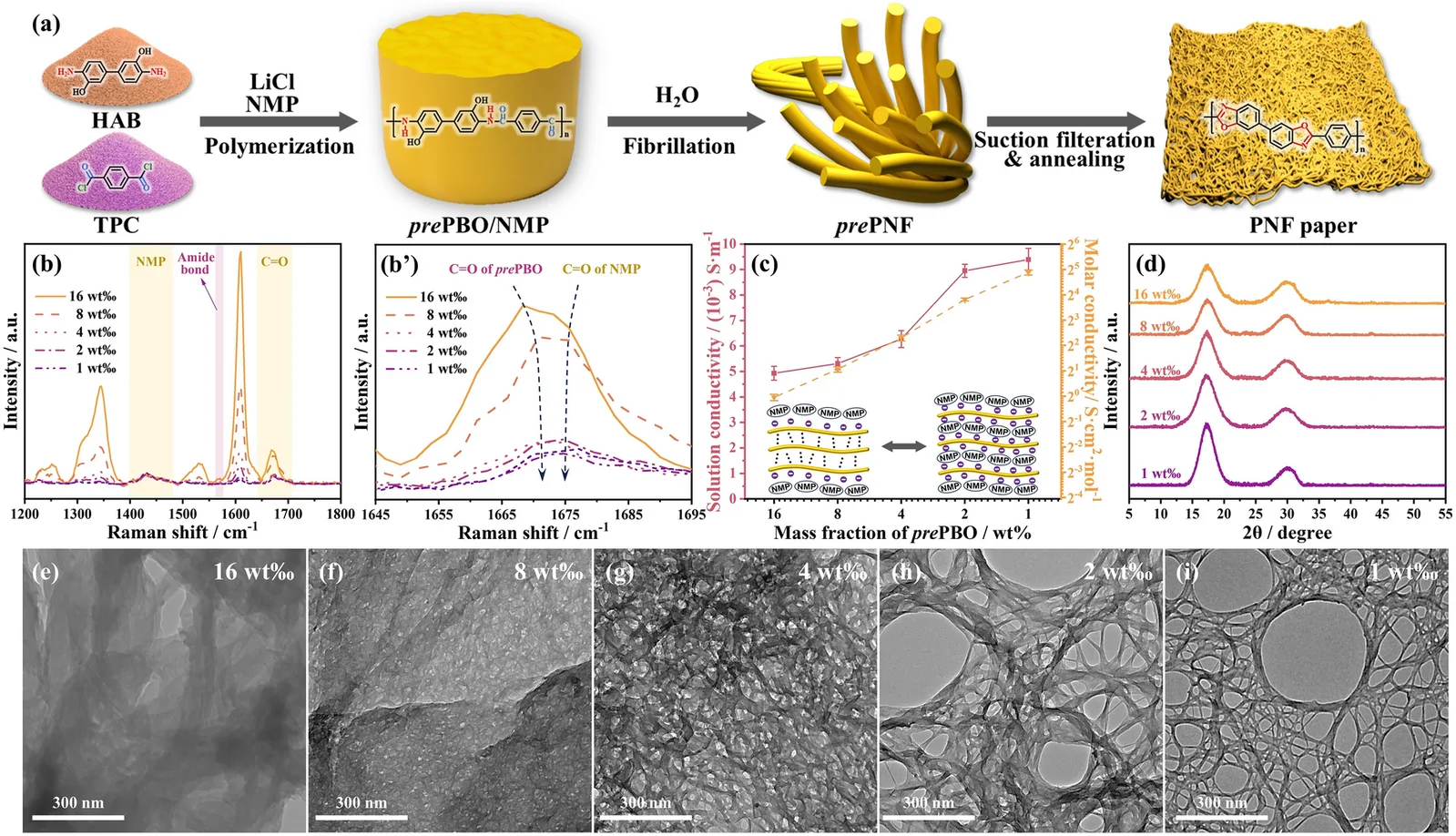
**a** Schematic diagram of preparing PNF paper via the down-top strategy; **b-b’** Raman spectra and **c** conductivity of the *pre*PBO solutions with different concentrations; **d** XRD curves and **e-i** TEM images of *pre*PNF prepared by *pre*PBO solutions of different concentration

Herein, a down-top strategy was developed to fabricate FG/PNF composite paper to address the aforementioned challenges. A PBO precursor (*pre*PBO) containing abundant active hydroxyl and amino groups was first synthesized via the polycondensation of 3,3’-dihydroxybenzidine and terephthaloyl chloride. The *pre*PBO solution was then protonated and dispersed in deionized water to form the PNF precursor (*pre*PNF, Fig. 1a), followed by uniformly mixing with FG. Consequently, FG/PNF composite paper was prepared via “vacuum-assisted filtration, hot-pressing, and annealing” processes. The hydroxyl and amino groups in *pre*PNF underwent oxazole cyclization during annealing to yield PNF. The optimal dispersion conditions of *pre*PNF were systematically investigated by Raman spectroscopy, Zeta potential, and transmission electron microscopy (TEM). Interactions between FG and PNF, along with composite paper structures, were characterized using X-ray photoelectron spectroscopy (XPS), X-ray diffraction (XRD), scanning electron microscopy (SEM), and interfacial peeling tests. Furthermore, the finite-difference time-domain (FDTD) and multiphysics finite element analysis (FEA) coupling analysis were applied to study the influence of down-top strategy prepared FG/PNF composite papers on wave-transparent and thermal conduction performances. This strategy enables large-scale production of highly wave-transparent and thermally conductive PNF-based composites, showing significant potential for applications in advanced electromagnetic devices such as radar antenna covers and electromagnetic windows.

## Experimental Section

The Materials, detailed experimental procedures, and characterizations are shown in Supporting Information.

## Results and Discussion

### Structure and Properties of PNF Paper Prepared by the Down-Top Strategy

*pre*PBO is first analyzed by FT-IR spectroscopy. Figure S1 shows characteristic peaks of amide groups at 3410, 1641, and 1495 cm^−1^, confirming the reaction between acyl chloride and amino groups [33]. As the concentration decreases, the *pre*PBO solution color shifts from golden yellow to transparent light yellow (Fig. S2). Besides, their Raman spectra (Fig. 1b) reveal reduced intensity of benzene ring and C-H vibrations at 1255, 1340, 1530, and 1610 cm^−1^ [34], primarily due to the decreased *pre*PBO content. The peak corresponding to pyrrolidine ring breathing mode of NMP at 1430 cm⁻^1^ [35] remains stable (Fig. S3a), which is due to the very high NMP content (> 970 wt‰). However, C = O stretching peak of NMP shifts from 1677 to 1674 cm⁻^1^, while C = O peak of *pre*PBO shifted from 1667 to 1672 cm^−1^. This is because high concentration of *pre*PBO in solution promotes intermolecular hydrogen bonding for *pre*PBO via hydroxyl/amide groups, whereas dilution increases hydrogen bonding between NMP and *pre*PBO, which reduces the hydrogen bond formation between *pre*PBO. Consequently, the length of C = O bond for NMP increased (red shift), while that of *pre*PBO decreased (blue shift) [16].

The amide bond peak at 1570 cm^−1^ weakens and vanishes with dilution (Fig. S3b), indicating LiCl-induced ionization of *pre*PBO amide groups, forming ionic species [36]. Conductivity and molar conductivity increase with decreasing concentration (Fig. 1c). When the concentration of *pre*PBO is 1 wt‰, the conductivity reaches the highest of 9.40 × 10^–3^ S m^−1^. This is mainly because, at higher *pr*ePBO concentrations, *pre*PBO molecules aggregate and cannot easily interact with LiCl and NMP to ionize. As *pre*PBO concentration decreases, abundant LiCl and NMP penetrated *pre*PBO molecules, disrupting intermolecular hydrogen bonds and dispersing *pre*PBO molecules. This increases the charge from ionization of hydroxyl and amide groups, thereby enhancing conductivity [34].

The XRD curves of *pre*PNF prepared from different concentrations exhibit the (110) characteristic peak at 17.3° that attributes to the nematic lyotropic liquid crystal (Fig. 1d), indicating that the nematic lyotropic liquid crystal structure of the nanofibers is retained during preparation [37–39]. Additionally, the intensity of this characteristic peak gradually increases as the concentration of the *pre*PBO solution decreased. This is because in low-concentration solutions, *pre*PBO molecules are separated from each other. Under deprotonation and shear forces, they are more easily oriented along the molecular chain direction to retain the nematic liquid crystal structure, thereby forming a long-range ordered crystal structure and forming nanofibers [40, 41].

The TEM images (Fig. 1e–i) reveal that at higher concentrations, the *pre*PBO dispersions prepared by deprotonation exhibit flake-like structures with little *pre*PNF present (Fig. 1e). As the *pre*PBO concentration decreases, voids gradually emerge within the *pre*PBO dispersions (Fig. 1f, g). When the concentration further reduces, the voids in *pre*PBO dispersions expand, facilitating the formation of fibrous structures. At a concentration of 2 wt‰ (Fig. 1h), the *pre*PBO dispersion displays a dendritic structure, where *pre*PNF diameters range between 20 and 300 nm. Upon further dilution to 1 wt‰, the *pre*PNF diameters narrow to 20 ~ 60 nm (Fig. 1i). The FT-IR spectrum of annealed PNF paper (Fig. S4) shows characteristic peaks at 1610 and 1053 cm^−1^, assigned to the C = N stretching of oxazole rings and C–O–C vibrations, respectively. The absence of amide bond absorption peaks confirms the cyclization of *pre*PNF into PNF [33].

**Fig. 2:**
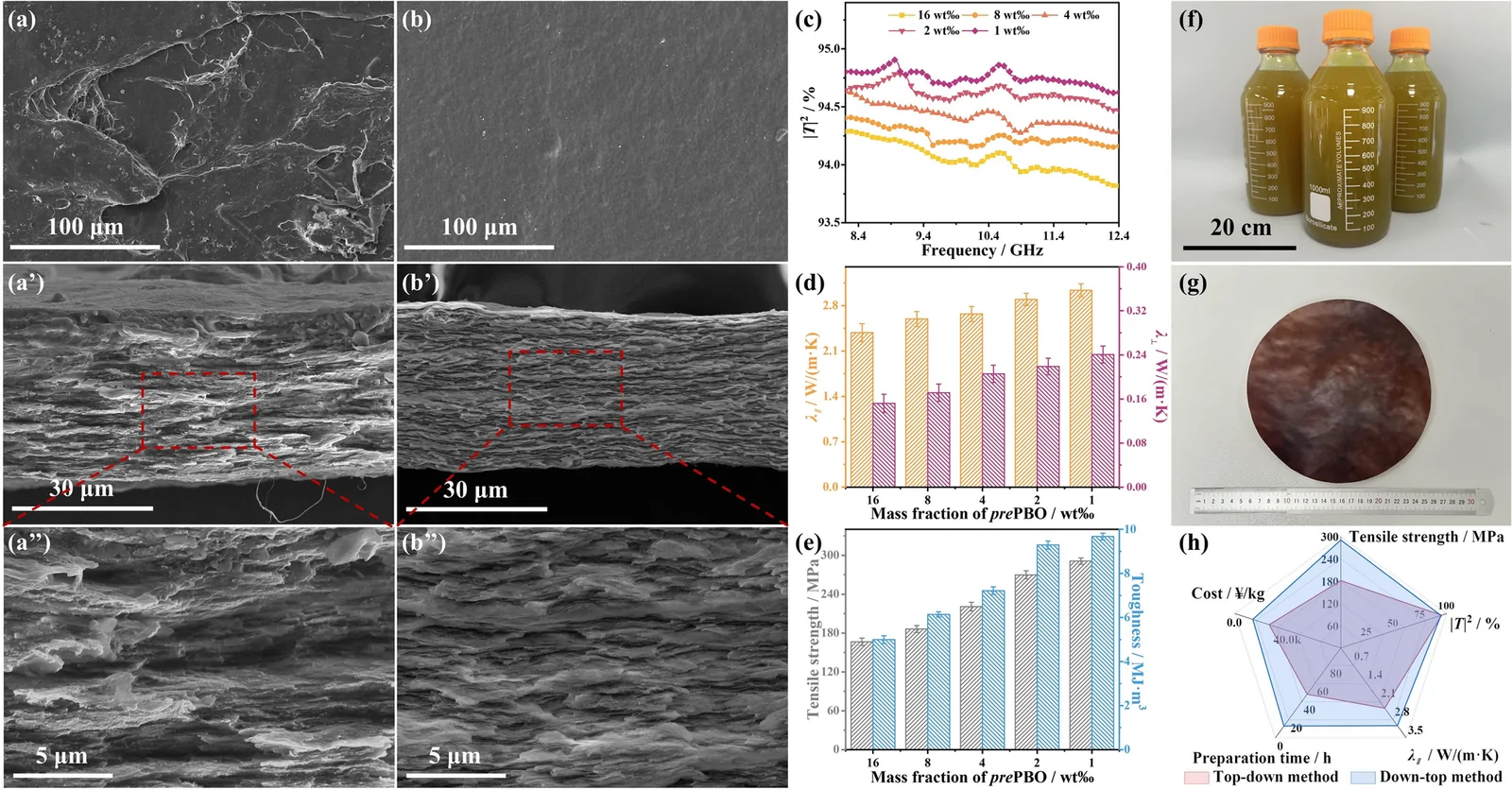
SEM images of the surface and fracture surface of PNF paper prepared with **a-a’’**16 wt‰ and **b-b’’** 1 wt‰ *pre*PBO solutions; **c** |*T*|^2^, **d** *λ*_*∥*_ and *λ*_⊥_, and **e** tensile properties of PNF papers; optical photographs of **f** *pre* PNF dispersion and **g** PNF paper prepared by the down-top strategy with 1 wt‰ *pre*PBO solution; **h** comparison of PNF paper prepared by top-down and down-top strategies

The surface and tensile fractures of PNF papers are characterized by SEM. For PNF paper prepared from 16 wt‰ *pre*PBO solution, exposed nanofiber with large diameters (> 500 nm) and numerous defects are observed on the surface (Fig. 2a-a**’’**). The tensile fracture exhibits interlayer slips along the Z-direction, with granular particles within the layered structure (Fig. 2a**’’**). This is attributed to the fact that 16 wt‰ *pre*PBO solution is hard to disperse by deprotonation to form small-diameter nanofibers (< 500 nm) but generates flake-like structures during preparation. During vacuum-assisted filtration, large-diameter nanofibers and flake structures disrupt the layered stacking process, hindering the formation of uniform nano-layers. This causes nanofiber exposed on surface and agglomerated into granular particles to form defects within the PNF paper. Additionally, these defects hinder interactions between PNF nano-layers, resulting in poor load transfer during stretching and ultimately interlayer slip failure along the Z-direction [42]. In contrast, PNF paper prepared from 1 wt‰ *pre*PBO solution shows a more uniform surface (Fig. 2b). Its tensile fracture displays distinct layered structures with minimal Z-direction slip, and individual nanofibers are rarely observed (Fig. 2b’, b’’). This indicates that failure primarily occurs through the nanofiber fracture [34]. The uniform diameter distribution of nanofibers from 1 wt‰ *pre*PBO solution promotes ordered layered stacking during vacuum-assisted molding, forming dense and homogeneous nano-layers. This structure enhances interactions between nano-layers, restricting relative slip and structural damage. Consequently, tensile failure is dominated by nanofibers breakages.

PNF papers prepared from different *pre*PBO concentrations exhibit clear trends in dielectric behavior. As the *pre*PBO concentration decreases, both the *ε* and tan*δ* of PNF paper gradually decline (Fig. S5a, b), while the |*T*|^2^ progressively increases (Fig. 2c) [13]. At 10 GHz, PNF paper made with 1 wt‰ *pre*PBO solution shows optimal wave-transparent performance. Its *ε* and tan*δ* decrease to 2.41 and 0.053, respectively, from 2.54 and 0.073 for PNF paper made from 16 wt‰ *pre*PBO solution. Correspondingly, |*T*|^2^ rises from 94.0% to 94.7%. This improvement is ascribed to the dense and uniform structure with well-aligned crystalline domains in PNF paper made from 1 wt‰ *pre*PBO solution (Fig. S6). The ordered configuration restricts molecular chain orientation-disorientation in high-frequency electromagnetic fields, thereby reducing electromagnetic loss [43, 44]. Conversely, charges accumulate at the defects of PNF paper made from 16 wt‰ *pre*PBO solution, which induces interfacial polarization and cause a lower |*T*|^2^. Additionally, it exhibits less stable wave-transparency (Δ|*T*|^2^ = 0.9%) compared to the PNF paper made with 1 wt‰ *pre*PBO solution (Δ|*T*|^2^ = 0.3%).

Figure 2d shows the *λ*_*∥*_ and *λ*_⊥_ of different PNF papers. As the *pre*PBO concentration decreases, both *λ*_*∥*_ and *λ*_⊥_ gradually increase. PNF paper made from 1 wt‰ *pre*PBO solution exhibits the highest thermal conductivity. Its *λ*_*∥*_ and *λ*_⊥_ rise to 3.04 and 0.24 W m^−1^ K^−1^, respectively, from 2.38 and 0.15 W m^−1^ K^−1^ for the PNF paper prepared with 16 wt‰ *pre*PBO. This is because the dense and uniform structure of PNF paper from 1 wt‰ *pre*PBO effectively reduces interfacial thermal resistance between PNF layers and minimizes phonon scattering [45]. Additionally, thermal annealing enhances the regularity of PBO molecular chains. Therefore, the crystal plane at 17.3° shows improved crystallinity (Fig. S6), further boosting phonon propagation efficiency along the PBO molecular chains [4, 46, 47].

Figures S7 and 2e show the tensile properties of PNF papers. As the concentration of *pre*PBO decreases, the mechanical performance gradually improves. For instance, PNF paper made from 1 wt‰ *pre*PBO solution exhibits the highest tensile strength and toughness of 291.8 MPa and 9.6 MJ m^−3^, respectively, compared to 166.5 MPa and 4.9 MJ m^−3^ for the PNF paper from 16 wt‰ *pre*PBO solution. This is because the uniform and dense structure of nanofiber and PNF paper (Figs. 1i and 2b’, b”) made from 1 wt‰ *pre*PBO solution promotes efficient stress transfer between PNF layers. In contrast, the agglomerated flake-like structures and numerous defects of PNF paper from 16 wt‰ *pre*PBO solution (Fig. 2a’, a”) cause stress concentration during stretching, thus leading to premature failure. Considering the best overall performance, PNF paper made from 1 wt‰ *pre*PBO solution is selected for the following studies.

Figure 2f, g shows the ethanol dispersion of *pre*PNF prepared from 1 wt‰ *pre*PBO solution and a corresponding PNF paper with a diameter of approximately 20 cm, respectively. Compared to the PNF paper prepared via top-down strategy (Top-down PNF paper), PNF paper prepared via down-top strategy (Down-top PNF paper) exhibits comparable wave-transparent performance while displays higher tensile strength and in-plane thermal conductivity. Meanwhile, the preparation time (from 50 to 14 h) and raw material cost (from 25,750 to 13,165 ¥ kg^−1^, Tables S1-S2) are significantly reduced by the down-top strategy. These advances demonstrate the potential for mass production of integrated wave-transparent, thermally conductive, and load-bearing PNF papers.

### Structure of FG/PNF Composite Papers

**Fig. 3:**
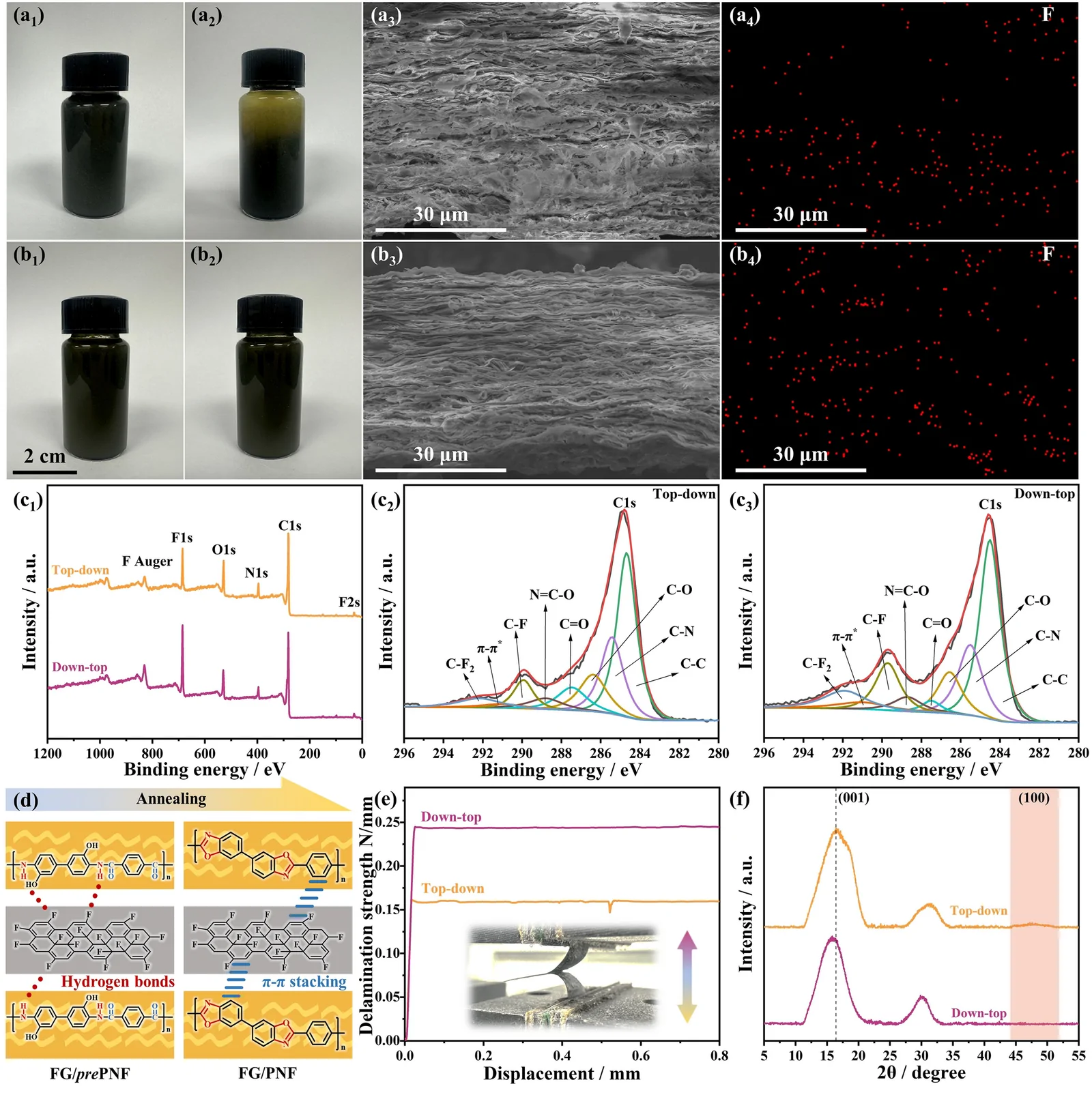
Optical photographs of the dispersion of **a**_**1**_**-a**_**2**_ FG/PNF and **b**_**1**_**-b**_**2**_ FG/*pre*PNF during the preparation process, SEM images of fracture surface of FG/PNF composite papers prepared by **a**_**3**_**-a**_**4**_ top-down and **b**_**3**_**-b**_**4**_ down-top strategies; **c**_**1**_**-c**_**3**_ XPS spectra of FG/PNF composite papers, **d** schematic diagram of the interaction between FG and nanofibers; **e** delamination peeling curves and **f** XRD curves of FG/PNF composite papers prepared by top-down and down-top strategies

Figure 3a_1_, b_1_ shows the ethanol dispersions of FG/PNF and FG/*pre*PNF after homogenizer mixing, respectively (the structure of FG please see the Supporting Information S3) [48, 49]. As observed, both dispersions appear uniformly dark green. After standing for 1 h, however, the FG/PNF dispersion shows phase separation as the upper layer turns yellow, while the lower layer remains dark green (Fig. 3a_2_). In contrast, the FG/*pre*PNF dispersion maintains stable by displaying a homogeneous dark green color (Fig. 3b_2_). This is because that FG and Top-down PNF exhibit strong chemical inertness and thus forms only weak hydrogen bonds and π-π interactions [50]. Additionally, Top-down PNF has a larger size and broader distribution (20 ~ 120 nm, Fig. S9), resulting in a smaller specific surface area. The poor compatibility between FG and PNF, combined with the higher density of FG, causes FG to sink and phase separation of the dispersion. Conversely, Down-top *pre*PNF contains abundant hydroxyl and amide groups on its surface. These groups form hydrogen bonds with FG, as confirmed by the increased binding energy of C-F_2_ (from 689.1 to 690.0 eV, Fig. S10) [51, 52]. This enhances their compatibility and stabilizes the dispersion, resulting in the structurally uniform FG/PNF composite paper. SEM images of tensile fractures further reveal structural differences. Top-down FG/PNF composite paper (Fig. 3a_3_) shows a loose structure with partial FG showing non-horizontal orientation. EDS mapping (Fig. 3a_4_) confirms F-element aggregation. In contrast, Down-top FG/PNF composite paper (Fig. 3b_3_) exhibits a dense structure with FG showing predominantly horizontal orientation. EDS mapping (Fig. 3b_4_) demonstrates uniform F-element distribution. These differences arise from the filtration process. For Top-down FG/PNF composite paper, the inert PBO molecules in PNF exhibit weak interactions with ethanol, accelerating the filtration speed (6.2 mL min^−1^, Fig. S11). This rapid process shortens the time for FG to rotate horizontally under gravity and fluid shear forces, locking the non-oriented structure [53]. For Down-top FG/*pre*PNF paper, hydrogen bonds in *pre*PNF, FG, and ethanol hinder ethanol removal during filtration. This reduces the filtration speed to 2.3 mL min^−1^ (Fig. S11) [54]. The slower rate extends the deposition time, enhancing structural denseness and improving FG orientation.

XPS was further employed to characterize the element content and interfacial interaction of FG/PNF composite papers (Fig. 3c_1_). Both kinds of composite papers show characteristic peaks at 31, 285, 398, 532, 686, and 831 eV, corresponding to F 2*s*, C 1*s*, N 1*s*, O 1*s*, F 1*s*, and F Auger peaks [55]. According to Tables S3 and S4, the Down-top FG/PNF composite paper exhibits higher F content, primarily due to the stable dispersion minimizing FG loss during its filtration process. In the C 1*s* spectra (Fig. 3c_2_, c_3_, Table S4) [56, 57], the C = O peak (287.5 eV) area decreases while the π-π interaction peak (291.0 eV) area increases. This is primarily due to the fact that carboxyl groups are generated during acid dissolution in Top-down PNF, whereas Down-top PNF generates no C = O groups during the preparation process. The minor C = O content originates from residual carboxyl groups on FG. Additionally, the loose structure of Top-down FG/PNF composite paper hinders π-π interactions between PNF-PNF and PNF-FG [58]. In contrast, the Down-top FG/PNF composite paper has a dense structure. After thermal annealing, hydroxyl and amide groups in *pre*PNF convert to oxazole rings to form PNF (Fig. 3d), increasing the π-π interactions between PNF and FG.

Delamination peeling tests (Fig. 3e) further demonstrate the interaction between the PNF and FG in FG/PNF composite papers [59]. The stress–strain curve of Top-down FG/PNF composite paper during peeling initially rises and then plateaus, exhibiting a low average plateau stress (0.15 N mm^−1^) with significant fluctuation. This is because FG aggregation within the PNF matrix reduces contact area with PNF, and stress drops at defect sites during peeling, resulting in weak interactions. In contrast, Down-top FG/PNF composite paper shows a higher average plateau stress (0.24 N mm^−1^) with stable peeling force, indicating enhanced compatibility and effective interfacial interactions between FG and PNF. These are consistent with XPS findings.

Figure 3f shows the XRD curves of FG/PNF composite papers. The Top-down FG/PNF composite paper exhibits diffraction peaks at 16.3° and 47.5°, corresponding to the (001) plane of the highly fluorinated hexagonal system in FG and the (100) plane formed by in-plane C–C bonds. In contrast, the (001) diffraction peak of the Down-top FG/PNF composite paper shifts from 16.3° to 15.8°, while the (100) diffraction peak nearly disappears. This is because FG sheets aligned horizontally and the F content increased at fluorinated regions, which expands the interplanar spacing and reduces the diffraction angle according to the Bragg equation [60, 61]. Additionally, horizontal FG orientation suppresses diffraction of the (100) plane, leading to a significant decrease of peak intensity [62, 63], further indicating that the Down-top FG/PNF composite paper achieves higher in-plane orientation.

### Wave-Transparent Performance of FG/PNF Composite Papers

**Fig. 4:**
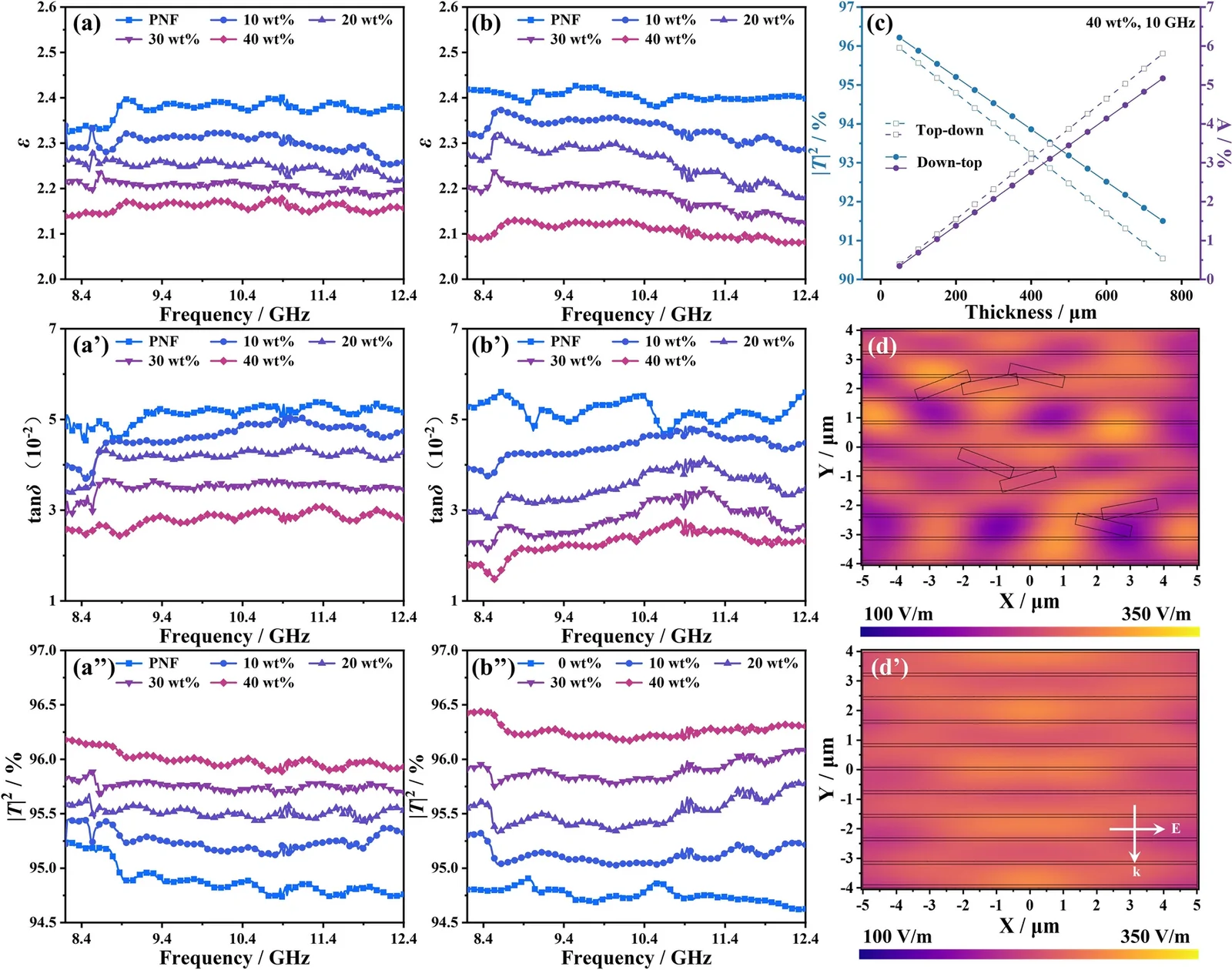
*ε*, tan*δ*, and |*T*|^2^ of FG/PNF composite papers prepared by **a-a’’** top-down and **b-b’’** down-top strategies; **c** |*T*|^2^ and A of FG/PNF composite papers with different thicknesses; near-field electric field distributions of FG/PNF composite papers prepared by **d** top-down and **d’** down-top strategies

Figure 4a, b shows the *ε*, tan*δ*, and |*T*|^2^ of FG/PNF composite papers prepared by top-down and down-top strategies. With increasing FG content, both *ε* and tan*δ* of the two types of composite papers gradually decrease, while the corresponding |*T*|^2^ gradually increases. When the frequency is 10 GHz and the mass fraction of FG is 40 wt%, the Down-top FG/PNF composite paper exhibits the lowest *ε* of 2.12 and tan*δ* of 0.023, corresponding to the highest |*T*|^2^ of 96.3%. This performance is superior to that of the pure Down-top PNF paper at the same frequency. Additionally, compared to the Top-down FG/PNF composite paper (*ε* = 2.17, tan*δ* = 0.029, |*T*|^2^ = 95.9%), the Down-top FG/PNF composite paper demonstrates better wave-transparent performances. Furthermore, as the thickness of FG/PNF composite papers increases, their |*T*|^2^ significantly decreases and the absorption rate (A) markedly rises. The increase in A for the Down-top FG/PNF composite paper is significantly smaller than that of the Top-down FG/PNF composite paper (Fig. 4c).

This is because that the Down-top FG/PNF composite paper exhibits strong π-π interactions between FG and PNF (Fig. 3c_3_, e), which restricts the orientation and disorientation of PNF molecular chains in high-frequency electromagnetic fields, thus reducing energy dissipation. Additionally, compared to pure PNF paper, FG possesses lower *ε* (< 2.0). The mismatch of *ε* at FG/PNF interfaces causes electromagnetic wave scattering and reflection. In Top-down FG/PNF composite papers, FG agglomeration and poor horizontal alignment (perpendicular to electromagnetic wave incidence) lead to significant scattering losses at agglomerates and interfaces. These losses intensify with increasing thickness, substantially reducing |*T*|^2^. Conversely, the uniformly dispersed and structurally ordered FG in Down-top FG/PNF composite paper aligned perpendicular to wave incidence, which minimizes such scattering and endows superior wave-transparent properties. To further investigate loss mechanisms, finite-difference time-domain (FDTD) simulations are applied to visualize electric field distributions of both types of FG/PNF composite papers at 10 GHz (Fig. 4d, d’, Table S5, Figs. S12 and S13), where E and k denote electric field vector and wave vector direction, respectively. As observed, both pure PNF papers show uniform electric fields with slight decay along propagation depth. However, the Top-down FG/PNF composite paper exhibits pronounced field attenuation (purple regions indicating strong dissipation) due to filler agglomeration and misalignment, resulting in an average field intensity of 218.4 V m^−1^ at Y = −4 μm. In contrast, the structurally ordered Down-top FG/PNF composite paper displays minimal field reduction (230.2 V m^−1^ at Y = −4 μm), confirming enhanced electromagnetic wave penetration.

### Thermal Conductivity of FG/PNF Composite Papers

**Fig. 5:**
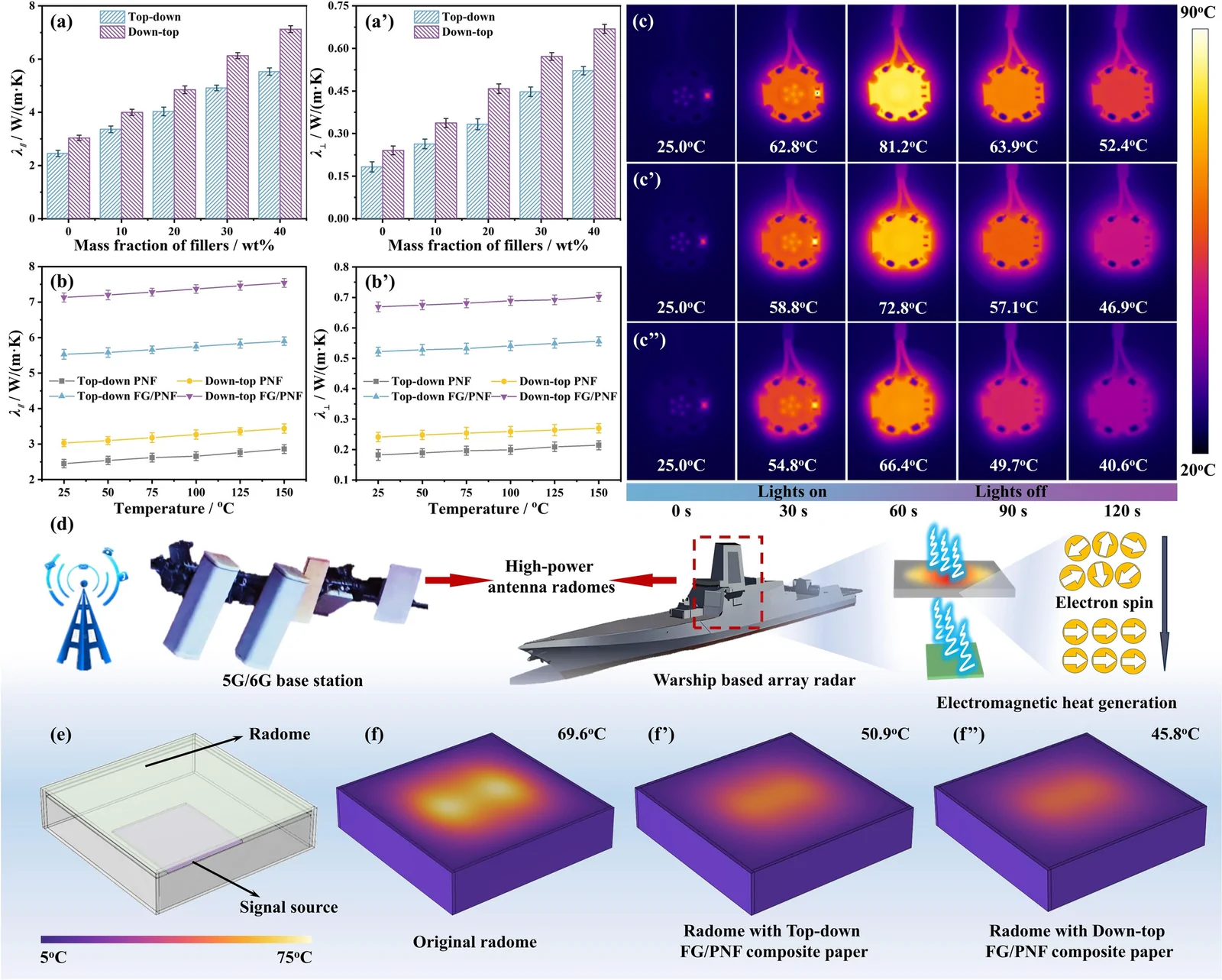
*λ*_*∥*_ and *λ*_⊥_ at **a-a’** room temperature and **b-b’** varied temperatures of FG/PNF composite papers prepared by top-down and down-top strategies; infrared thermal images of **c** original LED and LED with FG/PNF composite papers prepared by **c’** top-down and **c’’** down-top strategies; **d** potential applications of high-power antenna radomes and schematic diagram of electromagnetic heat generation; **e** FEA model and results of **f** original radome and radome with FG/PNF composite papers prepared by **f’** top-down and **f’’** down-top strategies

The *λ*_*∥*_ and *λ*_⊥_ of Top-down and Down-top FG/PNF composite papers are shown in Fig. 5a, a**’**. At room temperature, pure Down-top PNF paper exhibits *λ*_*∥*_ and *λ*_⊥_ of 3.04 and 0.24 W m^−1^ K^−1^, respectively, higher than those of pure Top-down PNF paper (*λ*_*∥*_ = 2.46 W m^−1^ K^−1^, *λ*_⊥_ = 0.18 W m^−1^ K^−1^. Meanwhile, both types of FG/PNF composite papers show increased *λ*_*∥*_ and *λ*_⊥_ with rising FG content, primarily due to the enhanced thermal conduction pathways formed by FG within the PNF matrix. Notably, Down-top FG/PNF composite paper demonstrates superior thermal conductivity to Top-down FG/PNF composite papers at the same FG loading. When the mass fraction of FG is 40 wt%, the Down-top FG/PNF composite paper achieves *λ*_*∥*_ and *λ*_⊥_ of 7.13 and 0.67 W m^−1^ K^−1^, respectively, representing 134.5% and 179.2% increase compared to pure Down-top PNF paper, and also significantly exceed the *λ*_*∥*_ (5.52 W m^−1^ K^−1^) and *λ*_⊥_ (0.52 W m^−1^ K^−1^) of Top-down FG/PNF composite paper.

Clearly, pure Down-top PNF paper exhibits higher intrinsic thermal conductivity than pure Top-down PNF paper. The Down-top FG/PNF composite paper also achieves greater enhancement in in-plane thermal conductivity compared to Top-down FG/PNF composite paper. This is because hydroxyl and amino groups generated by acid degradation during Top-down PNF preparation only partially cover the fiber surface (Fig. S10), hindering effective binding with FG and uniform dispersion. During vacuum-assisted filtration, FG tends to separate from PNF and agglomerate (Fig. 3a_1_-a_4_), obstructing the formation of continuous FG-FG thermal conduction paths and resulting in lower thermal conductivity of Top-down FG/PNF composite papers. Conversely, *pre*PNF in Down-top processing contains abundant hydroxyl groups and amide bonds, forming stable hydrogen bonds with FG that enhance dispersion. This facilitates self-assembly into structurally uniform FG/PNF composite papers with highly aligned FG in-plane during filtration. Thermal annealing preserves this oriented structure (Fig. 3b_1_-b_4_, 3f) [64], enabling efficient thermal pathway construction. Simultaneously, the higher density and stronger *π*-*π* interactions in Down-top FG/PNF composite papers (Table S4, Fig. 3f) yield superior through-plane thermal conductivity [65, 66]. Fitting experimental *λ*_*∥*_ and *λ*_⊥_ values of both types of composite papers using the modified Hashin–Shtrikman model (Fig. S14) [67] reveals lower interfacial thermal resistance (*R*_c_*) in Down-top FG/PNF composite papers: in-plane *R*_c_* (0.1828) and through-plane *R*_c_* (0.1655) versus Top-down FG/PNF composite papers’ in-plane *R*_c_* (0.1857) and through-plane *R*_c_* (0.1678). This confirms that enhanced FG-PNF interactions reduces interfacial thermal resistance, thereby improving thermal conductivity.

Additionally, both the *λ*_*∥*_ and *λ*_⊥_ of pure PNF paper and FG/PNF composite papers increase slightly with rising temperature (25 ~ 150 °C, Fig. 5b, b’). This occurs mainly because elevated temperatures accelerate phonon transport, enhancing thermal conduction in PNF papers and FG/PNF composite papers[68–70]. Figures 5c, c’ and S15 show infrared thermal images and corresponding surface temperatures of an LED bulb (**c**) and the same bulb attached to Top-down (**c’**) or Down-top (**c’’**) FG/PNF composite papers as heat dissipation materials. When air serves as the heat dissipation material, the LED bulb heats up fastest, reaching 81.2 °C after 60 s. In contrast, the Down-top FG/PNF composite paper exhibits the slowest heating rate, limiting the LED surface temperature to 66.4 °C after 60 s, lower than that of the Top-down FG/PNF paper (72.8 °C). After switching off the power, the Down-top FG/PNF composite paper cools to the lowest temperature most rapidly (Fig. S15), confirming its superior heat dissipation capability, consistent with the aforementioned *λ* results.

In 5G/6G base stations and Warship based array radar, the high emission power of electromagnetic waves causes molecular chains within radome materials to undergo orientation-disorientation cycles, generating heat (Fig. 5d) [71]. To address this, multiphysics FEA is applied to analyze the performance of FG/PNF composite papers as thermal diffusion films in high-power radar radomes. The simulation model and parameters are shown in Fig. 5e and Table S6. High radar emission power induces significant electromagnetic wave loss within the radome, producing substantial heat. Proximity between the radome and radar antenna further causes multiple internal reflections of electromagnetic waves, accumulating heat. However, the low thermal conductivity of the polymer matrix impedes efficient heat dissipation, leading to localized heat accumulation and elevated radome surface temperatures (69.6 °C). When incorporating PNF paper (Fig. S16) or FG/PNF composite paper as thermal diffusion films into the radome, the surface temperatures of high-power radar radomes decrease. This is primarily because the highly thermally conductive PNF and its composite papers effectively transfer heat generated by high-power electromagnetic waves to the radome wall’s heat dissipation module, where heat exchanges with the external environment. The Down-top FG/PNF composite paper exhibits optimal heat dissipation performance, achieving a radome surface temperature of 45.8 °C. This is significantly lower than that of pure Down-top PNF paper (58.3 °C) and Top-down FG/PNF composite paper (50.9 °C). Such thermal management extends the radome service life and reduces its detectability by infrared radar systems. Simultaneously, lower radome temperatures enhance wave-transparent performance and reduces energy consumption of radar system. Considering the relatively excellent performance, FG/PNF composite paper with 40 wt% FG made from the down-top strategy is selected for following investigations.

### Mechanical/Heat Resistance and Hydrophobic Properties of FG/PNF Composite Papers

**Fig. 6:**
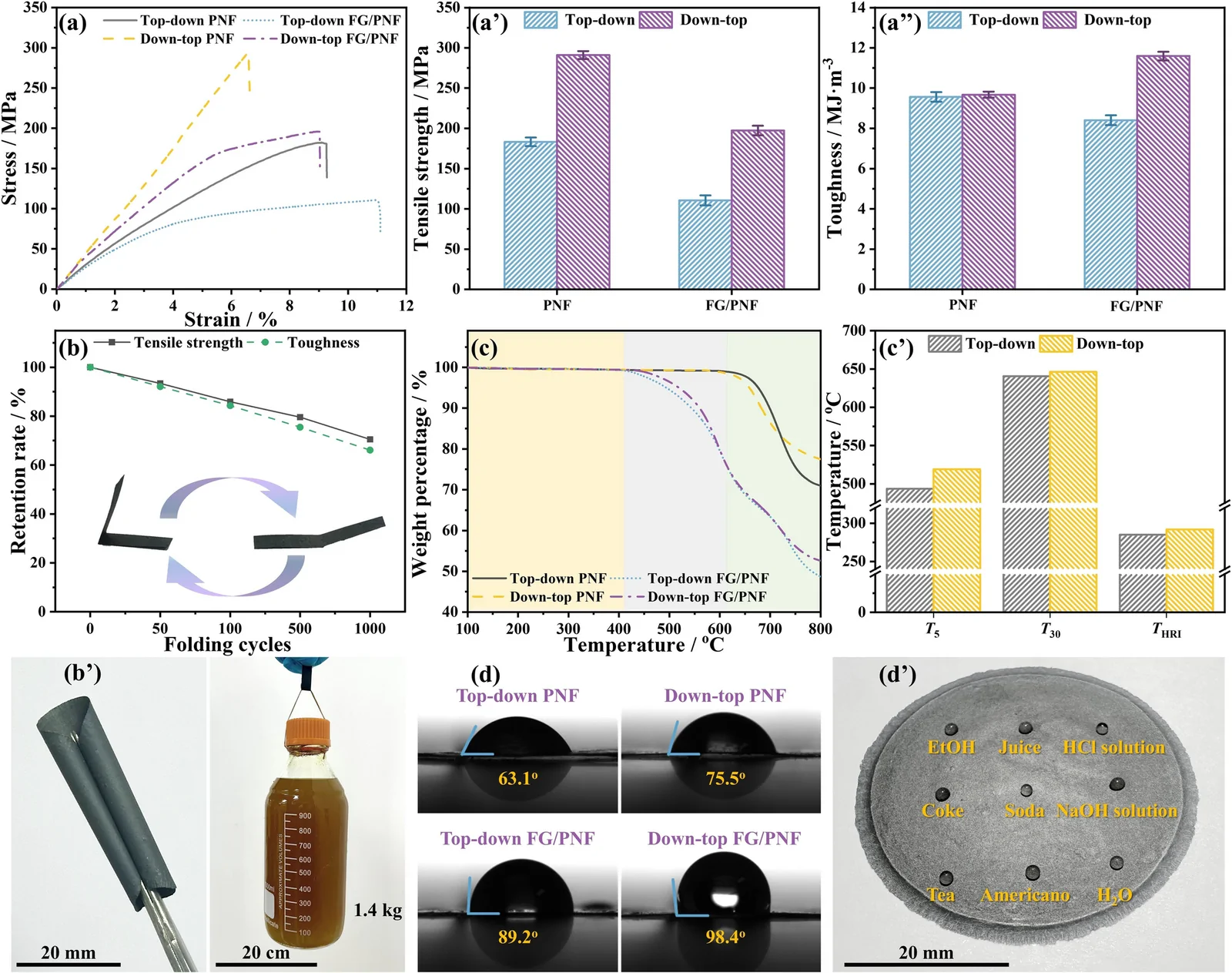
**a** Stress–strain curves, **a’** tensile strength, and **a’’** toughness of PNF papers and FG/PNF composite papers prepared by top-down and down-top strategies; **b** tensile strength and toughness retention and **b’** optical photographs of FG/PNF composite paper prepared by the down-top strategy; **c** TGA curves and **c’** thermal parameters, **d** water contact angles of pure PNF paper and FG/PNF composite papers prepared by top-down and down-top strategies; **c’** optical photographs of different liquids on the surface of FG/PNF composite paper prepared by the down-top strategy

Figure 6a-a**’’** shows the stress–strain curves and corresponding tensile strength/toughness of pure PNF papers and FG/PNF composite papers prepared by Top-down and Down-top strategies. As observed, the Down-top pure PNF paper shows a higher tensile strength than the Top-down pure PNF paper. This is because the tensile fracture of the Top-down PNF paper shows obvious interlayer slip and nanofibers extraction in the Z-direction (Fig. S17), where the fracture is mainly attributed to the failure of interlayer or nanofibers interactions. In contrast, the main reason for the tensile fracture failure of the Down-top PNF paper is the fracture of nanofibers (Fig. 2b**’**), which requires a relatively high force, endowing it with a higher tensile strength. After introducing FG, the tensile strength of both FG/PNF composite papers decreases compared to the pure PNF paper. This is primarily due to reduced PNF-PNF interactions caused by FG incorporation, weakening the stress transfer capability of FG/PNF composite papers. The Down-top FG/PNF composite paper exhibits significantly higher tensile strength (197.4 MPa) than the Top-down FG/PNF composite paper (110.6 MPa). Simultaneously, the Down-top FG/PNF composite paper also demonstrates optimal toughness (11.6 MJ m^−3^). This is mainly because the Down-top FG/PNF composite paper possesses a denser structure and stronger *π*-*π* interactions between FG and PNF, facilitating efficient load transfer. Additionally, the horizontally aligned and uniformly distributed FG within the composite paper effectively mitigates crack propagation during stretching, resulting in superior toughness [72, 73]. From Fig. 6b, after 1000 folding cycles, the Down-top FG/PNF composite paper retains over 65% of its original tensile strength and toughness. Furthermore, the composite paper can stand a 1.4 kg of *pre*PNF dispersion bottle. Moreover, the composite paper could also be curled, confirming excellent tensile strength and flexibility.

Figure 6c, c**’** displays the TGA curves and related parameters of pure PNF papers and FG/PNF composite papers. The Down-top PNF paper exhibits a slightly lower initial decomposition temperature than the Top-down PNF paper. Nonetheless, its decomposition temperature still exceeds 600 °C, indicating outstanding thermal stability. This phenomenon is mainly due to the symmetrical molecular structure of Top-down PNF which is conducive to the formation of long-range conjugated systems with more uniformly distributed electron cloud that requires higher energy for the thermal breaking of chemical bonds. Additionally, the Down-top PNF paper achieves higher char residue due to its lower O/N content and relatively higher C atomic concentration in the chemical structure. Simultaneously, both FG/PNF composite papers show two-stage weight loss. The first stage occurs near 410 °C, primarily attributed to FG decomposition. The second stage occurs around 630 °C, mainly resulting from PNF carbonization and pyrolysis. The Down-top FG/PNF composite paper achieves a Thermal Resistance Index (*T*_HRI_) [74] of 291.5 °C, higher than that of the Top-down FG/PNF composite paper (285.1 °C). This is primarily attributed to stronger π-π interactions between FG and PNF in the Down-top FG/PNF composite paper, which restricts FG decomposition at elevated temperatures.

Contact angle test for water on pure PNF papers and FG/PNF composite papers (Fig. 6d) reveals that FG incorporation enhances hydrophobicity, as evidenced by larger contact angles. This improvement is primarily attributed to uniform F-atom coverage on FG surfaces, which effectively blocks water contact with the composite papers [75]. Furthermore, both pure Down-top PNF paper and Down-top FG/PNF composite paper exhibit larger contact angles than their Top-down counterparts. The Down-top FG/PNF composite paper achieves a contact angle of 98.4°, higher than that of the Top-down FG/PNF composite paper (89.2°). This is because Top-down PNF tends to generate amino and carboxyl groups, whereas thermal annealing converts hydroxyl and amide bonds into oxazole rings in Down-top PNF. Consequently, Down-top PNF and its composite paper possess fewer surface-active groups (Fig. 3c_2_, c_3_), reducing hydrogen bonding with water. This results in poorer water spreading and larger contact angles on Down-top samples. Additionally, deionized water, Americano, and tea solutions form beaded droplets on the Down-top FG/PNF composite paper surface without spreading **(**Fig. 6e), further confirming its hydrophobic properties.

## Conclusions

Raman spectroscopy, Zeta potential, XRD, TEM, and SEM analyses demonstrate that protonating 1 wt‰ *pre*PBO solution under fluid shear via the Down-top strategy produces uniformly sized *pre*PNF. After thermal annealing, the resulting PNF paper exhibits excellent intrinsic wave-transparent and thermal conduction performances. This approach enables large-scale production of PNF paper with high efficiency. The hydroxyl and amino groups in *pre*PNF enhance the stability of the FG/*pre*PNF dispersion. Following thermal annealing, *π*-*π* interactions between PNF and FG improve their compatibility. The FG/PNF composite papers display outstanding wave-transparent and thermal conduction performance. When the mass fraction of FG is 40 wt%, the FG/PNF composite paper achieves a |*T*|^2^ of 96.3% (10 GHz), with *λ*_*∥*_ and *λ*_⊥_ reaching 7.13 and 0.67 W m^−1^ K^−1^, respectively. These values surpass those of the Top-down FG/PNF composite paper (|*T*|^2^ = 95.9% at 10 GHz; *λ*_*∥*_ = 5.52 W m^−1^ K^−1^, *λ*_⊥_ = 0.52 W m^−1^ K^−1^) and pure PNF paper (|*T*|^2^ = 94.7% at 10 GHz; *λ*_*∥*_ = 3.04 W m^−1^ K^−1^, *λ*_⊥_ = 0.24 W m^−1^ K^−1^). Simultaneously, this Down-top FG/PNF composite paper demonstrates superior mechanical properties, with tensile strength reaching 197.4 MPa and toughness of 11.6 MJ m^−3^.

## Supplementary Information

Below is the link to the electronic supplementary material.

Supplementary file1 (DOCX 2537 KB)
