# Supplementary material for: Down-Top Strategy Engineered Large-Scale Fluorographene/PBO Nanofibers Composite Papers with Excellent Wave-Transparent Performance and Thermal Conductivity
Source: Nanomicro Lett. 2025 Aug 20;18:35. doi: 10.1007/s40820-025-01878-y (PMC12364796; doi:10.1007/s40820-025-01878-y)
Supplement: Supplementary file 1 — Supplementary file1 (DOCX 2537 KB) [file 40820_2025_1878_MOESM1_ESM.docx]

Supporting Information for

**Down-Top Strategy Engineered Large-Scale Fluorographene/PBO Nanofibers Composite Papers with Excellent Wave-Transparent Performance and Thermal Conductivity**

Yuhan Lin^1^, Lin Tang^2^, Mingshun Jia^1^, Mukun He^1^, Junliang Zhang^1,^ *, Yusheng Tang^1^, Junwei Gu^1,^ *

^1^Shaanxi Key Laboratory of Macromolecular Science and Technology, School of Chemistry and Chemical Engineering, Northwestern Polytechnical University, Xi’an 710072, P. R. China

^2^Chongqing Key Laboratory of Green Catalysis Materials and Technology, College of Chemistry, Chongqing Normal University, Chongqing 401331, P. R. China

*Corresponding authors: Email: [junliang.zhang@nwpu.edu.cn](mailto:junliang.zhang@nwpu.edu.cn) (Junliang Zhang); [gjw@nwpu.edu.cn](mailto:gjw@nwpu.edu.cn) & [nwpugjw@163.com](mailto:nwpugjw@163.com) (Junwei Gu)

**S1 Experimental Section**

**S1.1 Main materials**

3,3’-Dihydroxybenzidine (HAB, 99%) and terephthaloyl chloride (TPC, 99%) were purchased from Shanghai Aladdin Biochemical Technology Co., Ltd. (Shanghai, China). Anhydrous lithium chloride (LiCl, 99%), N-methyl pyrrolidone (NMP, 98%), fluorinated graphite (FGi, 10 μm), trifluoroacetic acid (99%), and methanesulfonic acid (98%) were purchased from Shanghai Macklin Biochemical Co., Ltd. (Shanghai, China). Poly(p-phenylene benzobisoxazole) (PBO) fiber, model Zylon HM, density 1.56 g/cm³, was purchased from Toyobo Co., Ltd. (Japan). Sodium hydroxide (NaOH, 98%), potassium hydroxide (KOH, 98%), and anhydrous ethanol (98%) were purchased from Guangdong Guanghua Sci-Tech Co., Ltd. (Guangdong, China).

**S1.2 Fabrication of PNF paper by down-top strategy**

LiCl (2.5 g) was dissolved in N-methyl pyrrolidone (NMP, 100 mL) within a 250 mL flask under nitrogen atmosphere. Subsequently, HAB (4.32 g, 20 mmol) and propylene oxide (3 mL) were introduced and dissolved, followed by the addition of TPC (4.06 g, 20 mmol). The reaction was allowed to proceed for 1 h in ice-bath under stirring, followed by 12 h at room temperature to afford the PBO precursor (*pre*PBO) solution. The *pre*PBO solutions with the concentration of *pre*PBO being 16 wt‰, 8 wt‰, 4 wt‰, 2 wt‰, and 1 wt‰ were obtained by diluting the original *pre*PBO solution with NMP. These solutions were then dispersed in deionized water under 18,000 rpm fluid shear to form *pre*PNF. The resulting *pre*PNF was washed repeatedly with deionized water to neutrality. Finally, an appropriate amount of *pre*PNF was dispersed in ethanol, and *pre*PNF paper was prepared by “suction filtration-assisted hot-pressing and annealing”. The *pre*PNF paper was hot-pressed and annealed under nitrogen atmosphere at 100^o^C for 1 h + 200 ^o^C for 1 h + 350 ^o^C for 1 h to yield PNF paper. The preparation process of PNF paper is shown in **Fig. 1a**.

**S1.3 Synthesis of fluorinated graphene (FG)**

FG was prepared from fluorinated graphite (FGi) via a three-step procedure: alkaline activation, solvent intercalation, and ultrasonic exfoliation. Specifically, FGi (1 g), NaOH (1.26 g), and KOH (1.74 g) were homogenized and reacted at 170^o^C for 1 h. The product was cooled to room temperature, washed with deionized water to neutrality, filtered, and dried. The alkaline-activated FGi was dispersed in NMP (200 mL) under mechanical stirring. The mixture underwent reflux at 60^o^C for 2 h to achieve complete intercalation of NMP into FGi interlayers. Subsequently, the FGi/NMP dispersion was ultrasonically treated for 3 h using a cell disruptor (630 W) to obtain the FG/NMP dispersion. Finally, FG was obtained by centrifugation, filtration, and vacuum drying.

**S1.4 Fabrication of FG/PNF composite paper via down-top strategy**

FG was dispersed in ethanol and sonicated to uniformity. The resulting dispersion was added to a *pre*PNF ethanol dispersion. The mixture was homogenized using a high-shear homogenizer at 18,000 rpm and then processed through “vacuum-assisted filtration, hot-pressing, and annealing” to obtain FG/PNF composite papers with FG mass fraction of 10 wt%, 20 wt%, 30 wt%, and 40 wt%, respectively.

For comparison, a control composite paper was prepared by the top-down strategy: PBO fibers were dissolved in a TFA/MSA mixed acid and dispersed in deionized water to yield PNF according to the procedure of our previous work [S1]. FG was then blended with the PNF dispersion to fabricate FG/PNF composite papers containing identical FG mass fractions to the down-top strategy.

**S1.5 Characterizations**

Fourier transform infrared (FT-IR) spectra of the samples were obtained on Bruker Tensor 27 equipment (Bruker Corp., Germany) to characterize the chemical groups. Raman spectra of the samples were tested by Alpha300R confocal microscope Raman spectrometer (WITec Co., Germany). X-ray diffraction (XRD) curves of the samples were obtained on D8 DISCOVER A25 XRD equipment (Bruker Co., Germany) equipped with a Co target radiation source with a scanning speed of 5^o^/min. X-ray photoelectron spectroscopy (XPS, Kratos Axis Ultra DLD, UK) was used to analyze the elemental composition and binding energy variations of samples. Zeta potential of *pre*PBO solutions with different concentrations was measured by a dynamic light scattering analyzer (Mastersizer 2000, UK). Transmission electron microscope (TEM) images of the nanofibers were collected on a Talos F200X/TEM microscope (FEI Company, USA). Scanning electron microscopy (SEM) images were captured on a Verios G4 equipment (FEI Co., America). Dielectric properties of the samples at X band (8.2~12.4 GHz) were measured by an MS4644A Vector Network Analyzer instrument (Anritsu Corp., Japan), which applied the wave-guide method according to the standard of ASTM D5568-14. The in-plane thermal conductivity (*λ_∥_*) and through-plane thermal conductivity (*λ*_⊥_) of the composite papers were measured using a Hot Disk TPS2200 thermal constant analyzer (AB Co., Sweden). The *λ_∥_* was measured using the slab module and the *λ*_⊥_ was measured using the thin film module. For variable temperatures testing, the sample and the setup was placed in a convection oven integrated with the TPS2200 system. The oven temperature was raised to the test temperature and maintained for a while before the test was carried out. Infrared thermal images of the samples were obtained by Ti 300 infrared thermography (Fluke Co., USA). Mechanical properties of the samples were measured by a tensile testing machine (Guangdong Sitai Instrument Co., China) according to ASTM D5568-08. Thermogravimetric analyses (TGA) of the samples were carried out by STA 449F3 (NETZSCH C Corp., Germany) at 10^o^C/min (argon atmosphere), over the whole range of temperature (40~800 ^o^C). The dynamic contact angles of the samples were recorded by a contact angle meter (JY-82B Kruss DSA, Germany) according to ASTM D5725.

**S2 Supporting information for the structure and properties of PNF paper prepared by the down-top strategy**

**S2.1 Bragg’s formula for crystals**

$2dsin\theta=n\lambda$ (**S1**)

Where, *d* represents crystal plane spacing; *θ* represents the angle between the incident X-ray and the crystal plane; *λ* represents the wavelength of the incident X-ray; *n*: represents diffraction series.

**S2.2** **Calculation method of wave transparent properties, simulation model parameters and results for wave-transparent properties of wave-transparent composite papers**

The relationship between dielectric properties (*ε* and tan*δ*) and wave-transparent performance (|*T*|^2^) of the PNF paper is shown as follow:

$A=\frac{2\pi d\varepsilon\tan\delta}{\lambda(\delta-\sin^{2} \theta)^{1/2}}$ (**S2**)

${|\Gamma|}^{2}\mathbf{=}{[\frac{(\varepsilon-\sin^{2} \theta)^{\frac{1}{2}}-\varepsilon\cos\theta}{(\varepsilon-\sin^{2} \theta)^{\frac{1}{2}}+\varepsilon\cos\theta}]}^{2}$ (**S3**)

$A+|{T|}^{2}+{|\Gamma|}^{2}=1$ (**S4**)

Where, *d* represents the thickness of paper (50 μm); *λ* represents the wave length of the electromagnetic waves; *θ* represents the angle of incidence on the surface of paper; *ε* and tan*δ* represents the dielectric constant and dielectric loss tangent of the paper, respectively.

**S2.3 Supporting figures and tables for the structure and properties of PNF paper prepared by the down-top strategy**


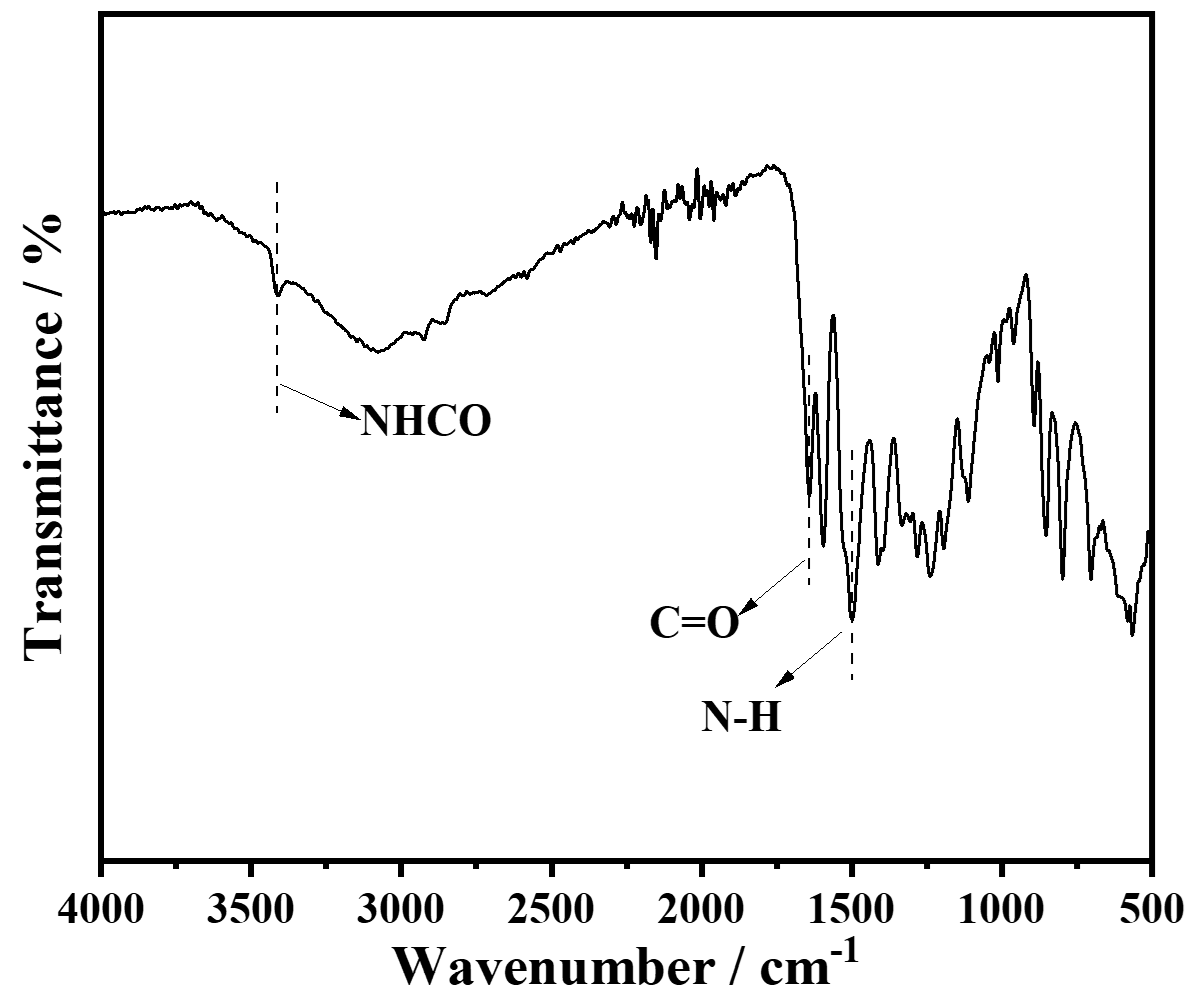


**Fig. S1** FT-IR spectrum of *pre*PBO


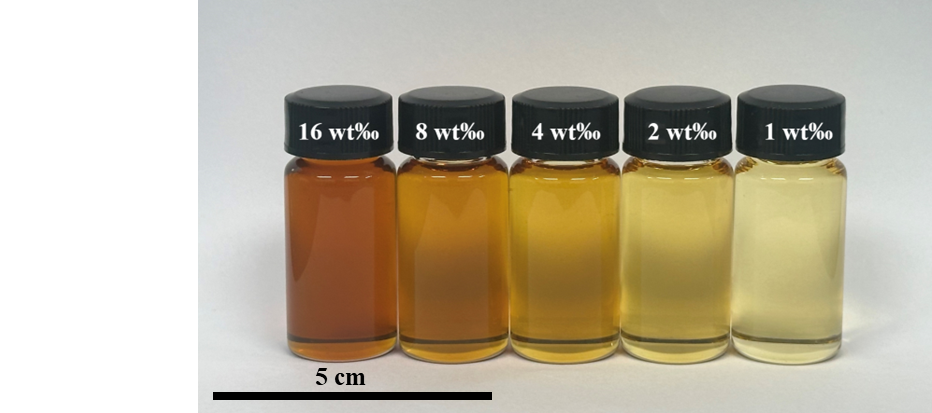


**Fig. S2** Optical photograph of *pre*PBO solutions with different concentrations


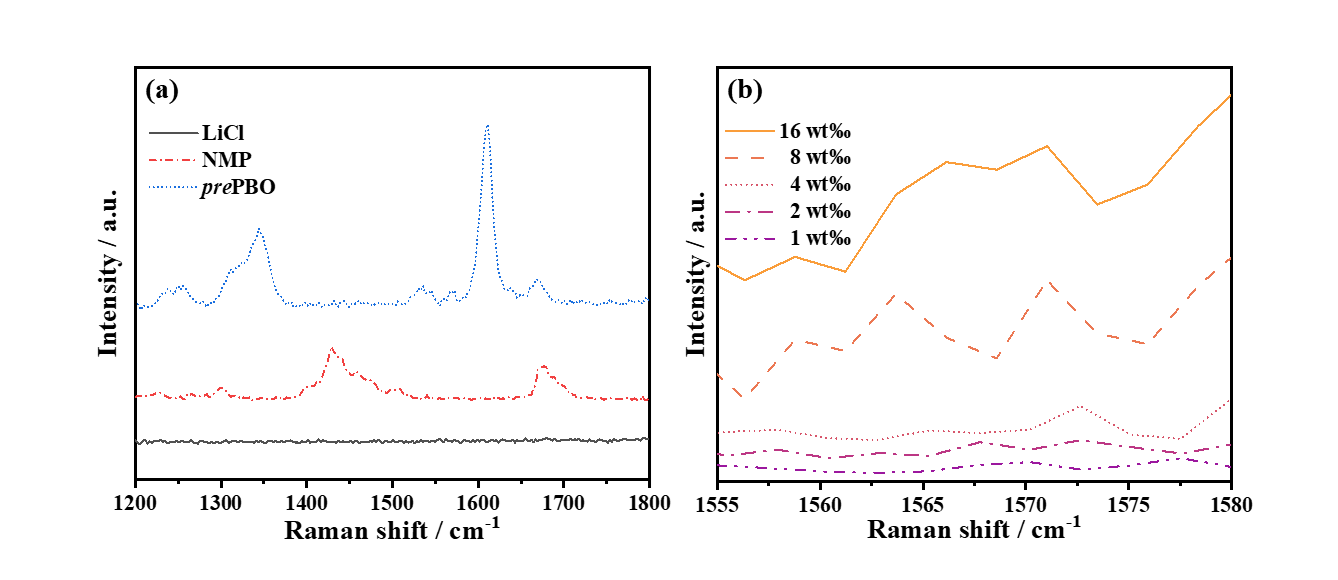


**Fig. S3** Raman spectra of LiCl, NMP, *pre*PBO (**a**) and *pre*PBO solutions (**b**)


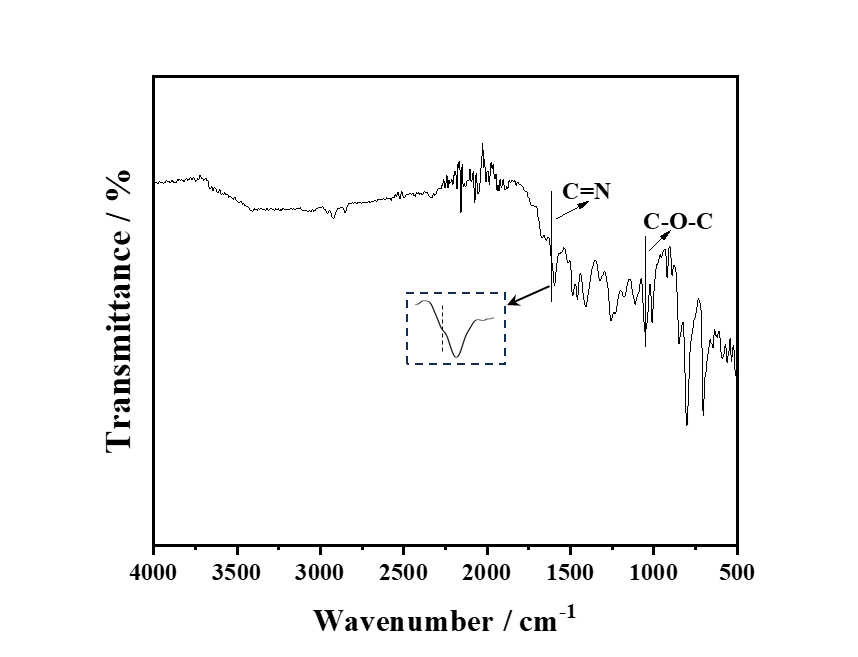


**Fig. S4** FT-IR spectrum of PNF paper after annealing by the down-top strategy


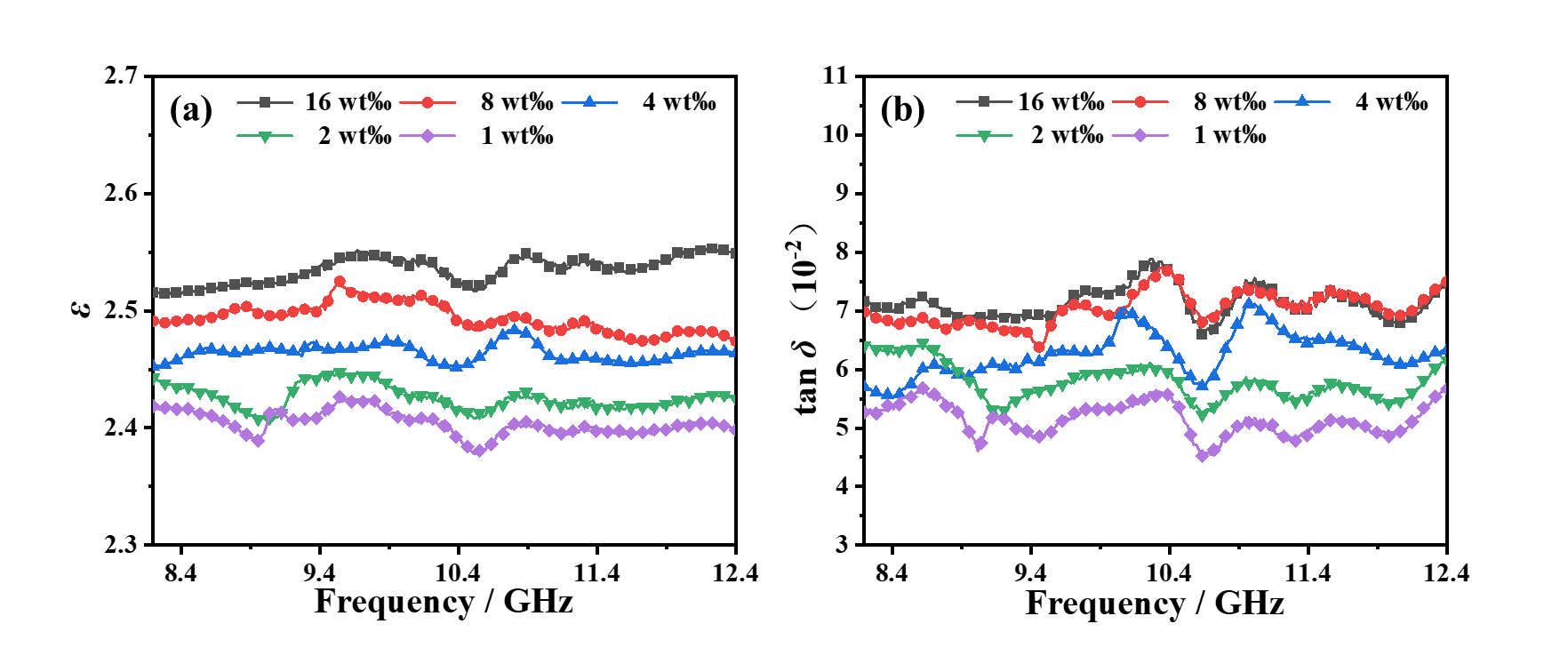


**Fig. S5** *ε* (**a**) and tan*δ* (**b**) of PNF papers prepared with different concentrations of *pre*PBO solutions


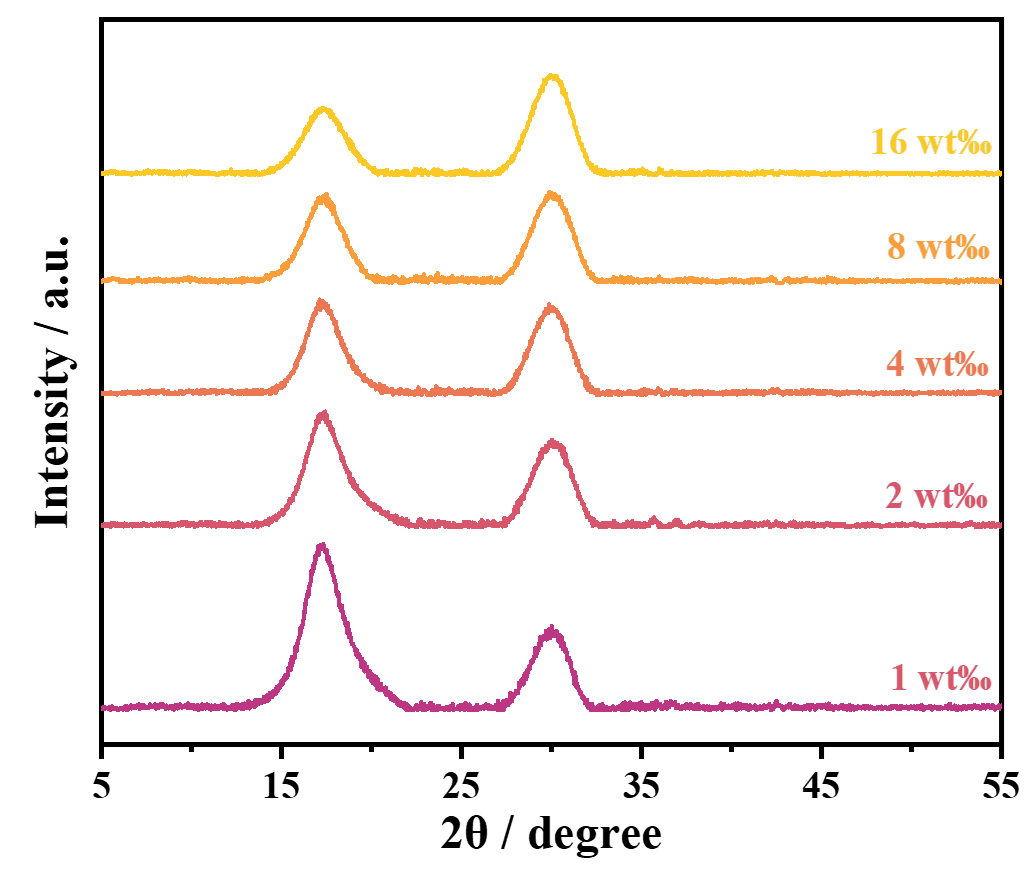


**Fig. S6** XRD curves of PNF papers prepared with different concentrations of *pre*PBO solutions


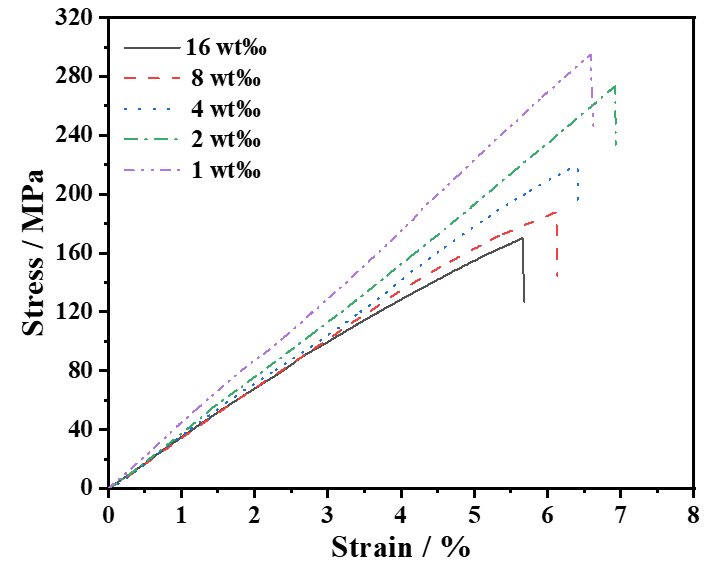


**Fig. S7** Stress-strain curves of PNF papers prepared with different concentrations of *pre*PBO solutions

**Table S1** The raw material price for PNF paper prepared by top-down strategy for industrial preparation

| **Chemical** | **Purchase website** | **Price (¥/kg)** | **Dosage (kg)** | **Cost (¥)** |
| --- | --- | --- | --- | --- |
| **PBO fiber** | toyobo-global.com | 2500 | 1 | 2500 |
| **TFA** | 1688.com | 39 | 500 | 19500 |
| **MSA** | 1688.com | 2.5 | 500 | 1250 |
| **EtOH** | 1688.com | 5.0 | 500 | 2500 |
| **Total price** | | | | 25750 |

**Table S2** The raw material price for PNF paper prepared by down-top strategy for industrial preparation

| **Chemical** | **Purchase website** | **Price (¥/kg)** | **Dosage (kg)** | **Cost (¥)** |
| --- | --- | --- | --- | --- |
| **HAB** | 1688.com | 6000 | 0.516 | 3096 |
| **TPC** | 1688.com | 80 | 0.484 | 39 |
| **LiCl** | chemicalbook.com | 100 | 0.298 | 30 |
| **NMP** | 1688.com | 7.5 | 1000 | 7500 |
| **EtOH** | 1688.com | 5 | 500 | 2500 |
| **Total price** | | | | 13165 |

**S3** **Supporting information for the** **structure of FG/PNF composite papers**

**
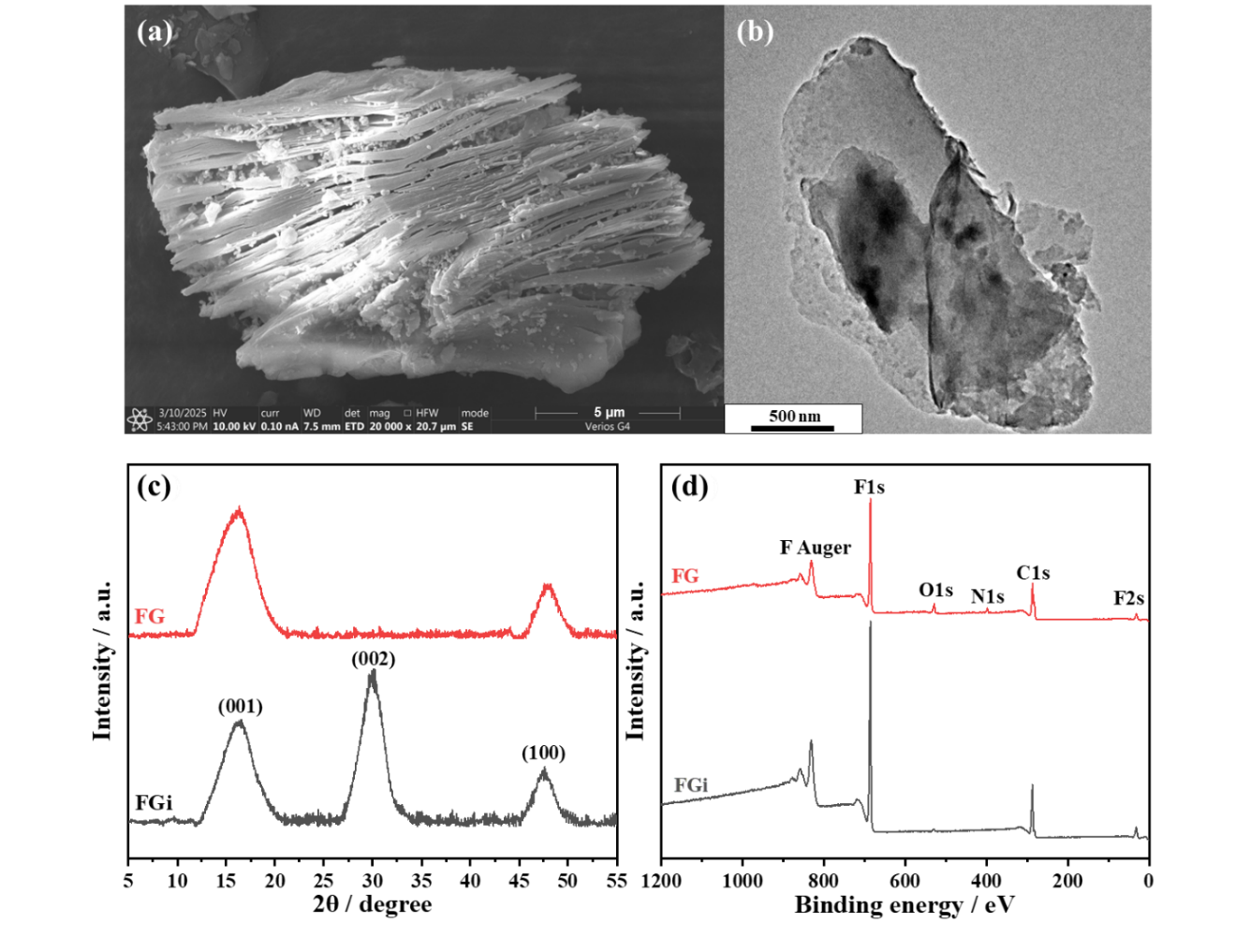
**

**Fig. S8** SEM image of FGi (**a**), TEM image of FG (**b)**, XRD (**c**) and XPS (**d**) spectra of FGi and FG

The fluorinated graphite (FGi) reveals an accordion-like multilayered structure as displayed by the SEM image (**Fig. S8a**). After the treatment of “alkaline activation-solvent intercalation, and ultrasonic exfoliation”, FG with a flaky structure was obtained (**Fig. S8b**). The XRD curve of FGi exhibits diffraction peaks at 16.3^o^, 29.1^o^, and 47.5^o^, corresponding to the (001), (002), and (100) crystalline planes, respectively [S2]. In contrast, the (002) peak associated with layer stacking disappears in FG, while the (001) and (100) peaks remain essentially unchanged (**Fig. S8c**) [S3]. Additionally, the F/C ratio of FG is nearly unchanged (**Table S3**) with the N and O content slightly increasing (**Fig. S8d**). These results indicate that N and O containing functional groups are introduced into FG while the core crystal structure of FGi is preserved during exfoliation.


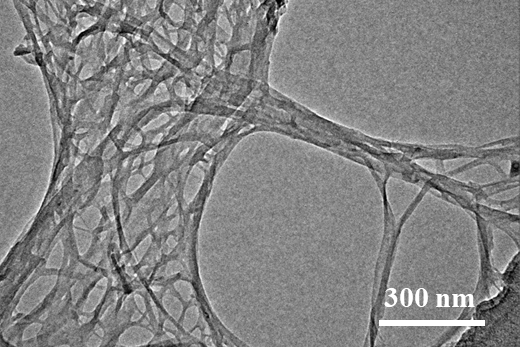


**Fig. S9** TEM image of PNF prepared by top-down strategy


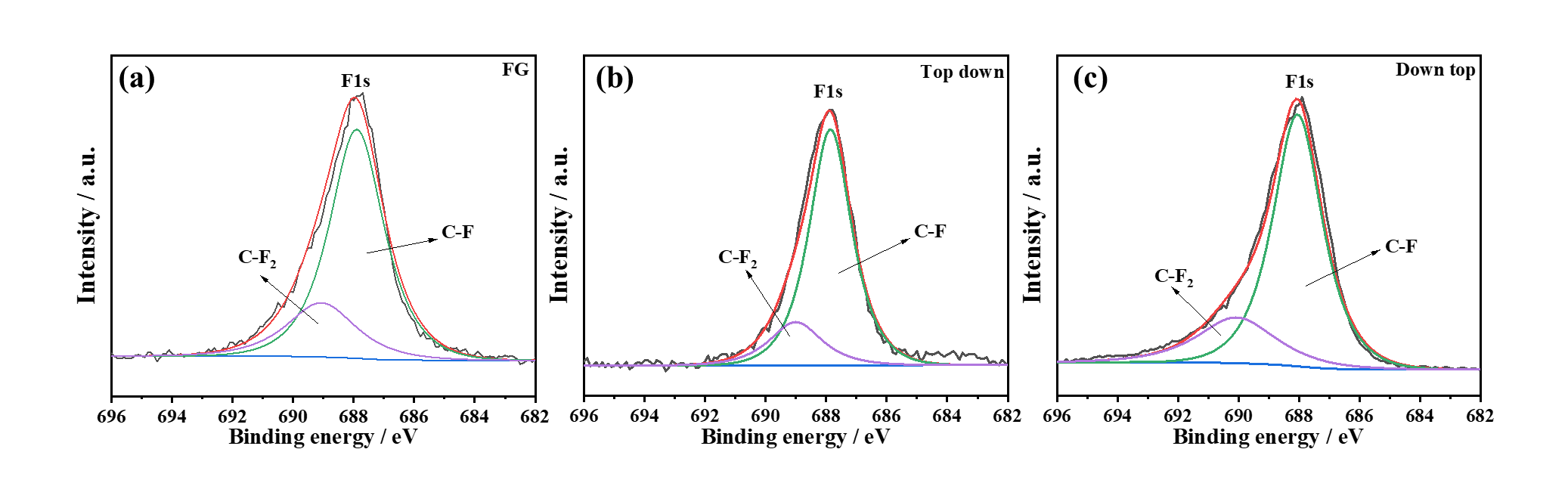


**Fig. S10** High-resolution XPS spectra of F1s of FG (**a**), FG/PNF (**b**), and FG/*pre*PNF (**c**)


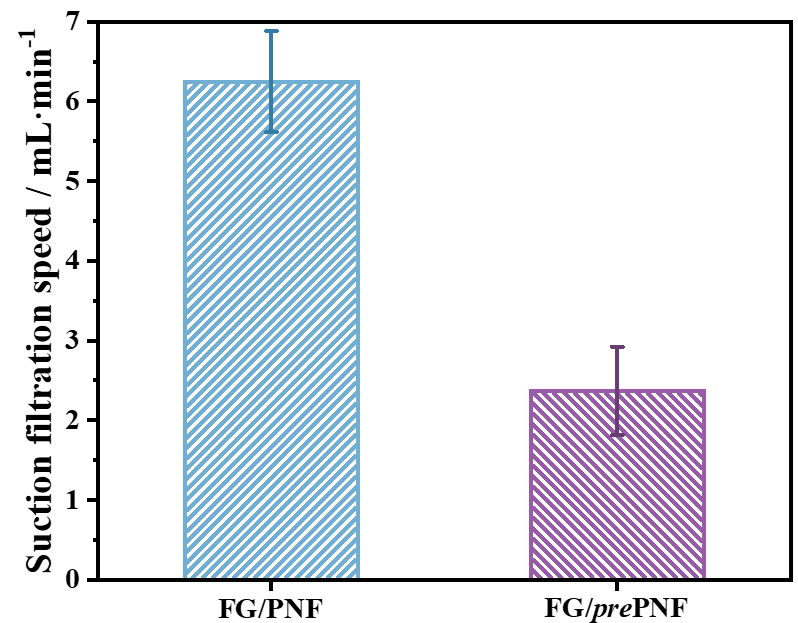


**Fig. S11** Suction filtration speeds of FG/PNF and FG/*pre*PNF

**Table S3** Molar fraction (%) of surface elements for FGi and FG

| **Sample** | **C** | **N** | **O** | **F** |
| --- | --- | --- | --- | --- |
| **FGi** | 74.4 | 4.1 | 8.7 | 12.8 |
| **FG** | 72.0 | 5.7 | 9.8 | 12.5 |

**Table S4** The proportion of each chemical bond in the C1s peak for FG/PNF composite papers prepared by top-down and down-top strategies (%)

| **Strategy** | **C-C** | **C-N** | **C-O** | **C=O** | **N=C-O** | **C-F** | **π-π^*^** | **C-F_2_** |
| --- | --- | --- | --- | --- | --- | --- | --- | --- |
| **Top-down** | 40.7 | 20.6 | 12.5 | 5.3 | 4.1 | 8.7 | 1.6 | 6.5 |
| **Down-top** | 42.1 | 18.8 | 12.3 | 1.7 | 4.0 | 10.3 | 3.4 | 7.4 |

**S4 Supporting information for Wave-transparent property of FG/PNF composite papers**

**S4.1 Finite-Difference Time-Domain (FDTD) simulation model building process for PNF papers and FG/PNF composite papers**

The electromagnetic wave scattering behaviors of papers were simulated by COMSOL Multiphysics 6.1 software. The geometric models were established according to the real situation. The model parameters are shown in **Table S5**.

**Table S5** Simulation model parameters for the papers

| **Model parameters** | | **Values** |
| --- | --- | --- |
| Port type | Rectangular port | |
| Port module | 1 | |
| Port mode type | Transverse electric | |
| Input power (W/m) | 10 | |
| Electromagnetic wave frequency (GHz) | 10 | |
| Distance between input port and surface of paper (μm) | 5 | |

**S4.2** **Supporting figures of wave-transparent property for FG/PNF composite papers**


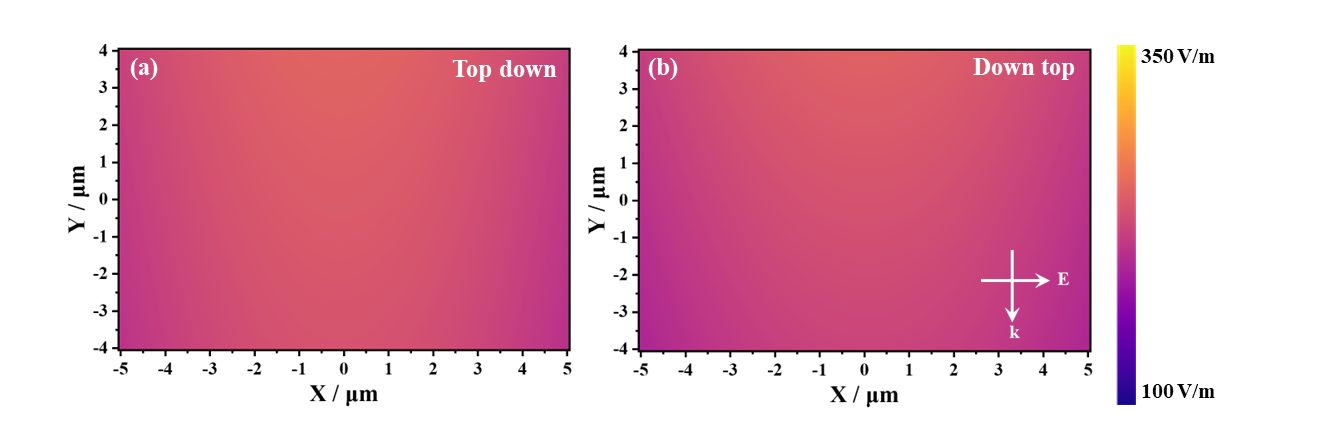


**Fig. S12** Near-field electric field distributions of PNF papers prepared by top-down (**a**) and down-top strategies (**b**)


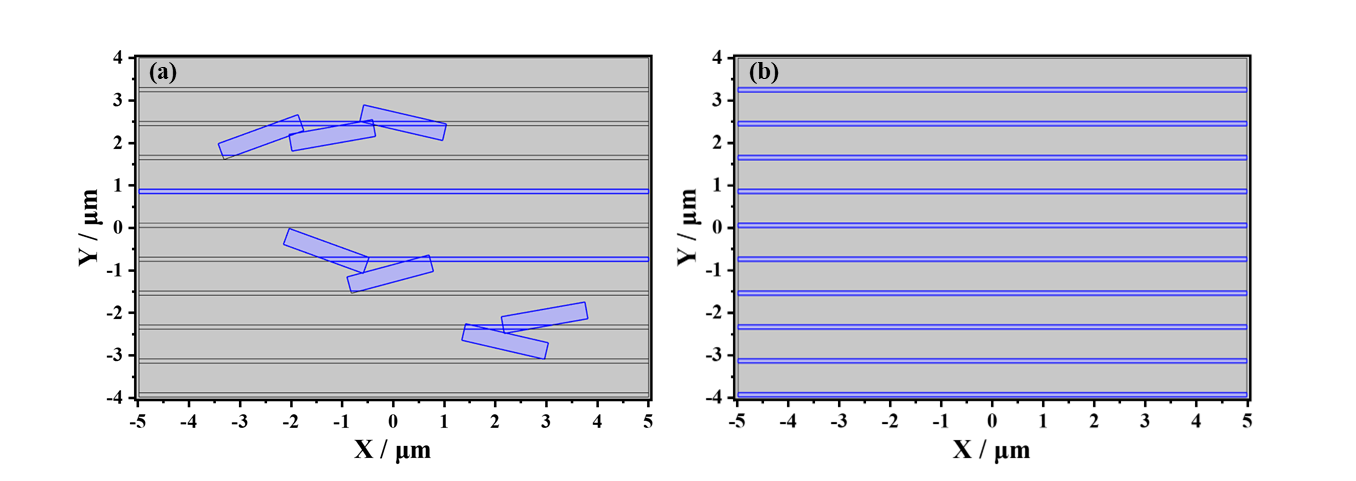


**Fig. S13** The simulation structure of FG/PNF composite paper prepared by top-down (**a**) and down-top (**b**) strategies (blue for FG and gray for PNF)

**S5 Supporting information for thermal conductivity of FG/PNF composite papers**

**S5.1 Calculation method for Hashin-Shtrikman model**

$K=13.3347exp（-13.2701R_{c}^{*}）$ (**S5**)

$\lambda_{c}^{*}=\frac{\lambda_{c}}{\lambda_{p}}=\frac{1+\sum_{i=1}^{n} E_{i}V_{i}}{1\mathbf{-}K\sum_{i=1}^{n} E_{i}V_{i}}$ (**S6**)

$E_{i}=\frac{\frac{\lambda_{f}}{\lambda_{p}}-1}{\frac{\lambda_{f}}{\lambda_{p}}+(D-1)}$ (**S7**)

$V_{i}=\frac{1}{1+\frac{1-\rho_{i}\omega t}{\rho_{p}\omega t}}$ (**S8**)

Where *λ*_p_, *λ*_f_ and *λ*_c_ represent the *λ* of matrix, fillers and composites; *V*_i_ represents the volume fraction of filler, *ρ*_i_ and *ρ*_p_ represent the density of composites and matrix, respectively. *n* is the number of species for fillers (n = 1 for this work) and *D* = 3. *K* is a coefficient, which is related to the total thermal resistance of composites.

**S5.2 Multiphysics simulation model building process for PNF papers and FG/PNF composite papers**

The radome surface temperatures were simulated by COMSOL Multiphysics 6.1 software. The model parameters are shown in **Table S6**.

**Table S6** Simulation model parameters for the Radome

| **Model parameters** | | **Values** |
| --- | --- | --- |
| Shape  parameters of radome | Fiber-reinforced resin composite  radome size (2 layers) | 200 mm×200 mm×2 mm |
|  | Heat dissipation film size (sandwiched between 2 layers of fiber-reinforced resin composite) | 200 mm×200 mm×0.3 mm |
|  | Distance between radome and antenna | 40 mm |
| Material parameters of radome | Fiber-reinforced resin composite | *λ*=1.0 W/(m·K); *ε*=3.0; tan*δ*=0.005 |
|  | Heat dissipation film | Based on the experimental test |
| Transmitting antenna parameter | Mouth surface size | 100 mm×100 mm |
|  | Mouth surface strength | Equal amplitude distribution |
|  | Angle of polarization | 0^o^ |
|  | Input power | 100 W/m |

**S5.3 Supporting figures of thermal conductivity for FG/PNF composite papers**


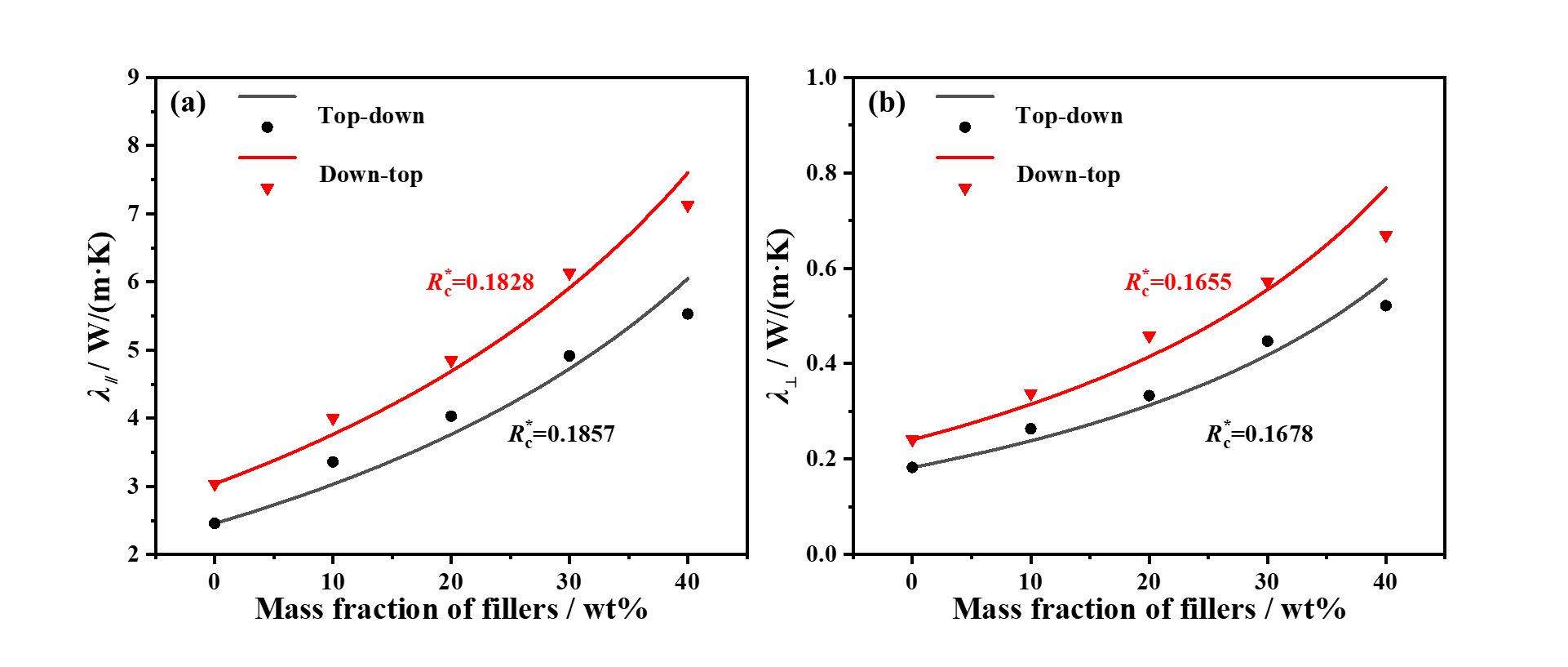


**Fig. S14** Fitting *λ_∥_* (**a**) and *λ*_⊥_ (**b**) of FG/PNF composite papers prepared by top-down and down-top strategies using modified Hashin-Shtrikman model

**Fig. S15** Temperature-time relationship curves of original LED and LED with FG/PNF composite papers prepared by top-down and down-top strategies


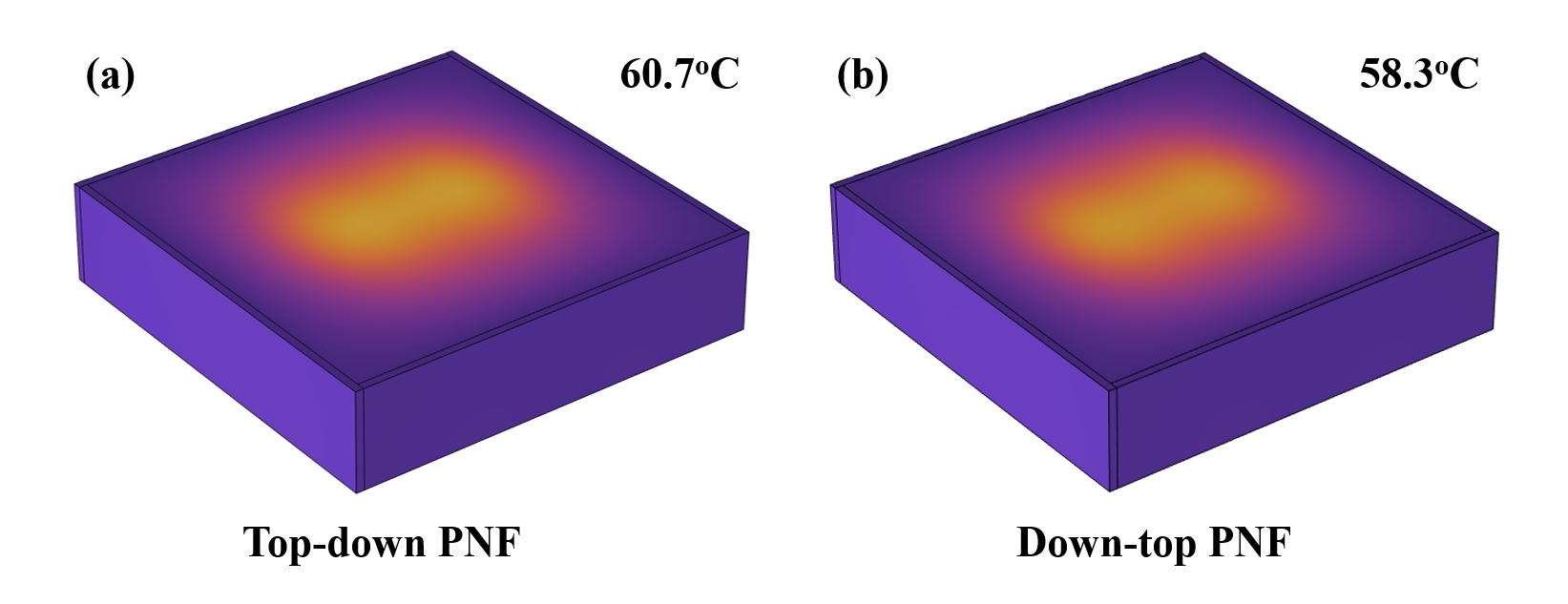


**Fig. S16** FES results of PNF papers prepared by top-down and down-top strategies

**S6 Supporting information for mechanical properties of FG/PNF composite papers**


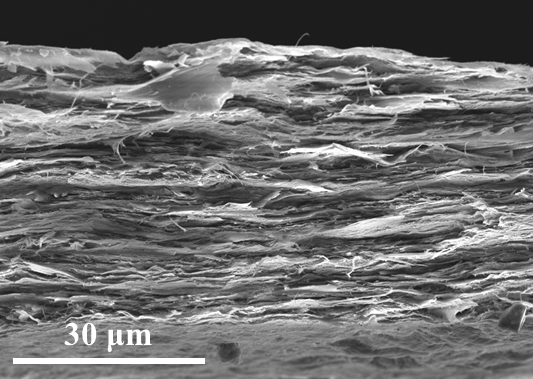


**Fig. S17** SEM image of tensile fracture for Top-down PNF paper

**Supplementary References**

1. Y. Lin, X. Fan, L. Tang, Y. Tang, J. Gu, Polysilsesquioxane-PBO wave-transparent composite paper with excellent mechanical properties and ultraviolet aging resistance. Adv. Fiber Mater. **5**(6), 2114–2126 (2023). <https://doi.org/10.1007/s42765-023-00327-y>
2. P. Gong, S. Ji, J. Wang, D. Dai, F. Wang, M. Tian, L. Zhang, F. Guo, Z. Liu. Fluorescence-switchable ultrasmall fluorinated graphene oxide with high near-infrared absorption for controlled and targeted drug delivery. Chem. Eng. J. **348**, 438-446 (2018). <https://www.sciencedirect.com/science/article/pii/S1385894718307666>
3. Wang, F, Liu, Z, Li, J, Huang, J, Fang, L et al, Lateral heterostructure formed by highly thermally conductive fluorinated graphene for efficient device thermal management. Adv. Sci. (Weinh) **11**(25), e2401586 (2024). <https://doi.org/10.1002/advs.202401586>
